# Inside out: microbiota dynamics during host-plant adaptation of whiteflies

**DOI:** 10.1038/s41396-019-0576-8

**Published:** 2020-01-02

**Authors:** Diego Santos-Garcia, Natividad Mestre-Rincon, Einat Zchori-Fein, Shai Morin

**Affiliations:** 1 Department of Entomology, The Hebrew University of Jerusalem, P.O. Box 12, 76100 Rehovot, Israel; 2 Department of Entomology, Newe-Ya’ar Research Center, ARO, Ramat-Yishai, Israel

**Keywords:** Microbial ecology, Microbiome

## Abstract

While most insect herbivores are selective feeders, a small proportion of them feed on a wide range of plants. This polyphagous habit requires overcoming a remarkable array of defenses, which often necessitates an adaptation period. Efforts for understanding the mechanisms involved mostly focus on the insect’s phenotypic plasticity. Here, we hypothesized that the adaptation process might partially rely on transient associations with bacteria. To test this, we followed in a field-like experiment, the adaptation process of *Bemisia tabaci*, a generalist sap feeder, to pepper (a less-suitable host), after switching from watermelon (a suitable host). Amplicon sequencing of 16S rRNA transcripts from hundreds of dissected guts revealed the presence of active “core” and “transient” bacterial communities, dominated by the phyla Proteobacteria, Actinobacteria, and Firmicutes, and increasing differences between populations grown on watermelon and pepper. Insects grown on pepper for over two generations presented a significant increase in specific genera, mainly *Mycobacterium*, with a predicted enrichment in degradative pathways of xenobiotics and secondary metabolites. This result correlated with a significant increase in the insect’s survival on pepper. Taken together, our findings suggest that gut-associated bacteria can provide an additional flexible metabolic “tool-box” to generalist sap feeders for facilitating a quick host switching process.

## Introduction

Generalist insects are extraordinary in their ability to perceive diverse olfactory and gustatory cues, digest complex nitrogen and carbon compounds, and detoxify different secondary defense metabolites [1, 2]. Because in every host switch these insects are required to establish new temporal interactions and to overcome a new array of plant defenses in a short time, their adaptation to a new host is believed not to involve genotypic selection, but the production of different phenotypes with one given genotype [3]. One way this can be achieved is by phenotypic plasticity, a process that allows the production of different physiological, morphological, or behavioral phenotypes using the same genome [4]. For example, the well documented extensive rearrangement of the expression pattern of detoxification genes in generalist insect species that occurs shortly after host shifts, which likely induces the required physiological plasticity for mitigating the new host defensive chemistry [5]. Alternatively, insects are known for being able to establish a diverse array of symbiotic interactions with bacteria. Transient ones, especially those involving bacteria found on the insect’s surface or the gut lumen (termed ectosymbionts), can facilitate rapid transitions between phenotypes, which might be more adapted to new environmental conditions [6, 7].

The diversity of bacteria inhabiting the gut lumen of insects is determined by different factors including diet, environmental habitat, developmental stage, social behavior, and phylogeny of the host [8–10]. These bacteria are predicted to play a major role in insects host-plant adaptation, mainly because they are present in the organ where food is initially processed, and therefore, can respond relatively fast to environmental changes, such as the presence of phytotoxins in the insect diet. Also, gut-associated bacteria are in contact with free-living external bacteria and can acquire new genetic material, including detoxifying genes, or can even be replaced by new bacteria with different metabolic potential [7]. Our study focused on the possible contribution of gut-associated bacteria to the ability of the whitefly *Bemisia tabaci* (Hemiptera: Sternorrhyncha: Aleyrodoidea) to switch between host plants. *B. tabaci* is a phloem-feeding insect pest characterized by high fecundity, short developmental time, significant dispersal capability, and ability to use multiple plants as hosts [11]. Previous culture-independent approaches have reported that *B. tabaci*, as other sternorrhynchan insects (psyllids and aphids), presents low (if any) variety of gut-associated ectosymbionts [10, 12, 13]. This low diversity was mainly attributed to the sternorrhynchan simple gut morphology, the nearly microbial-free diet (phloem), the ability of the insects to digest the food quickly, and the presence of intracellular bacteria, also called endosymbionts, that might compete for the host resources [6, 12, 13]. All whiteflies, including *B. tabaci*, harbor an obligatory endosymbiont named *Portiera aleyrodidarum* and different facultative endosymbionts, including the genera *Hamiltonella*, *Arsenophonus*, *Rickettsia*, *Wolbachia*, and *Cardinium* [11, 14]. *Portiera* and *Hamiltonella/Arsenophonus* are always harbored inside specialized host cells (bacteriocytes) and complement their host’s diet [15, 16]. *Cardinium*, *Wolbachia*, and *Rickettsia* have different tissue tropisms [17], not including the gut lumen [18], and likely do not contribute to the diet complementation of their host [16, 19]. In addition, a meta-analysis of *B. tabaci* endosymbiotic communities and their host plants concluded that whiteflies’ endosymbionts seem not to be involved in plant adaptation [14]. In contrast to culture-independent approaches, several studies that used culture-dependent methods have clearly indicated that surface-sterilized whiteflies and their feces (“honeydew”) present a diverse array of bacterial species, suggesting the presence of gut microbiota [20, 21]. Such bacteria might be actively or passively acquired from microbial communities inhabiting plant surfaces or inner tissues [7]. Taking into account the transient and unpredictable nature of this environmentally-acquired gut microbiota, we hypothesized that under some temporal and spatial conditions (including bacterial community interactions), the gut-microbiota dynamics might contribute to the ability of *B. tabaci* to successfully switch between host plants, including cases in which the new host can be considered as a challenging, less-suitable one. To test this hypothesis, we conducted a multigeneration adaptation experiment, under field-like conditions, using suitable (watermelon) and less-suitable (pepper) host plants. Each generation, we monitored the active gut-associated bacterial communities by extracting total RNA from dissected gut samples. To reduce the number of 16S rRNA endosymbiotic sequences, and ameliorate their masking effect, we applied a PCR protocol that depleted these targets before conducting the final amplicon sequencing. In parallel, performance assays on the two host plants were conducted.

## Materials and methods

A detailed version of Materials and Methods, including its associated Figs. S8–S10 and Tables S2–S4, is available at the Supplementary Information.

### Insects, plants, and field experiment

Few thousands *Bemisia tabaci* adults were collected from two watermelon fields and transferred to rearing chambers, containing the same watermelon cultivar. The population was identified as the Middle East Asia Minor 1 species of *B. tabaci* and harbored the endosymbionts *Portiera*, *Hamiltonella*, and *Rickettsia*. The field-like experiment was conducted in two neighboring plots at the Hebrew University Experimental Farm. Pepper, a less-suitable host plant [22–24], was used as the “treatment” diet. Watermelon, a suitable host plant [25–27] and the original host of the collected population, was used as the “control” diet. The boosted *B. tabaci* field-derived colony was used as the starting population. Single watermelon/pepper plants were planted in buckets filled with in-site soil. Single plants were placed inside insect rearing tents. Every new generation, adults were collected, tents were cleaned, and new plants, with in-site soil, were placed in each tent before establishing the new generation. The tents allowed biotic (mainly microorganisms) and abiotic factors to pass freely from the surrounding environment to the inner plants. Each generation, newly emerged adults were transferred from the field experiment to a controlled greenhouse. Adult couples were left to oviposit on new watermelon or pepper plants, according to their field host plant (three leaves per plant, two plants), and egg-to-adult survival was recorded.

### Gut-enriched RNA samples, sequencing, and amplicons analysis

Because sequencing of total DNA extraction cannot distinguish between live (transcriptionally active) and quiescent or dead bacteria (nontranscriptionally active) composing the gut microbiota [28], total RNA instead of DNA was extracted from the gut samples. For each RNA sample in each generation, 12 females per tent were collected and surface sterilized. Then, their guts were dissected and pooled, homogenized with a bead-beater, and total RNA was extracted and reverse-transcribed to cDNA. Three specific blocking dual priming oligonucleotides (bDPOs) were designed in order to reduce *Portiera*, *Hamiltonella*, and *Rickettsia 16S* rRNAs PCR amplification. Amplicons were obtained using the 27F (V1) and 515R (V3) universal primers and the three bDPOs. Amplicons from cDNA samples, a mock community, and a negative control were sequenced by a MiSeq system (V3 chemistry, 2 × 300 base paired-reads) at the DNA Services Facility (University of Illinois, Chicago).

RAW reads were quality filtered, assembled, and clustered using USEARCH. Taxonomic classification of zero-radius operational taxonomic units (ZOTUs) was performed with mothur. Data preprocessing, library-size normalization, alpha and beta diversity, and differential abundance were conducted with phyloseq among other R packages. Core (ZOTUs present in all experimental groups), shared (ZOTUs consistently shared between experimental groups), and specific (ZOTUs consistently present in a single experimental group) were computed by averaging the abundance of the corresponding ZOTU and discarding any ZOTU that did not appear in at least two samples from the same experimental group under analysis (host plant and/or generation). Microbiomes metabolic potential were predicted with PICRUSt and HUManN. LEfSe was used to screen for significant differences in the inferred metabolic pathways.

## Results

### Sequencing results

From the 40 sequenced *B. tabaci* gut-enriched samples, 34 passed all quality checks (Table S1). The average RAW library size after the pair-ended assembly was 148,734 ± 72,014 SD (18,455–346,968). After the ZOTUs clustering, the average mapping percentage was 93% ± 11% SD, corresponding to an average mapped library size of 141,644 ± 70,126 SD (7651–331,540). Subsequent discarding of all three endosymbionts’ reads (*Portiera*, *Hamiltonella*, and *Rickettsia*) reduced the average library size to 20,659 ± 28,655 (3201–111,066). This gave a bDPOs average blocking efficiency of 18% ± 23% SD (2–100%). After discarding all potential contaminants (kits, environment, PCR, etc.), the average analyzed library size was further reduced to 15,070 ± 21,201 SD (1863–91,377). No significant correlations (confounding effects) were found between the cDNA amounts used for the PCRs, bDPOs blocking efficiency, library size, and diversity indices (Table S1). When only putative gut-associated bacteria were considered, the average number of ZOTUs per library was 133 ± 66.9 SD (48–320) with Shannon and Simpson richness indices averaging 3.32 ± 0.42 SD and 0.92 ± 0.03 SD, respectively. Samples with library sizes close to, or larger than, 5000 reads showed convergence in their sampling-effort curves, while those with smaller library sizes did not reach a plateau, indicating that not all the diversity was sampled (Fig. S1).

### *B. tabaci’s* gut-associated bacteria α diversity

**Fig. 1:**
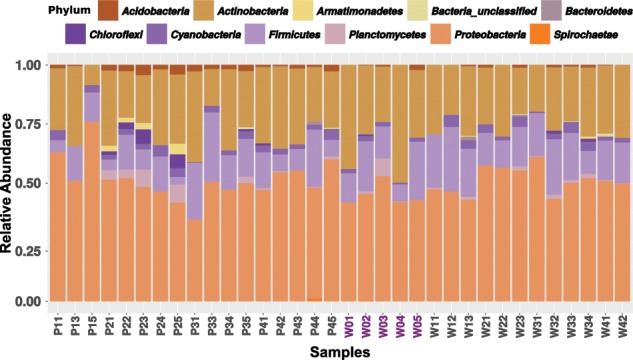
The alpha diversity of *B. tabaci* gut-enriched samples. The bar plot shows the relative abundance of the bacterial ZOTUs, collapsed at the phylum level. Samples starting by P and W denote samples from whiteflies reared on pepper or watermelon, respectively. Numbers denote the generation. The starting population samples (W0) are highlighted in purple.

**Fig. 2:**
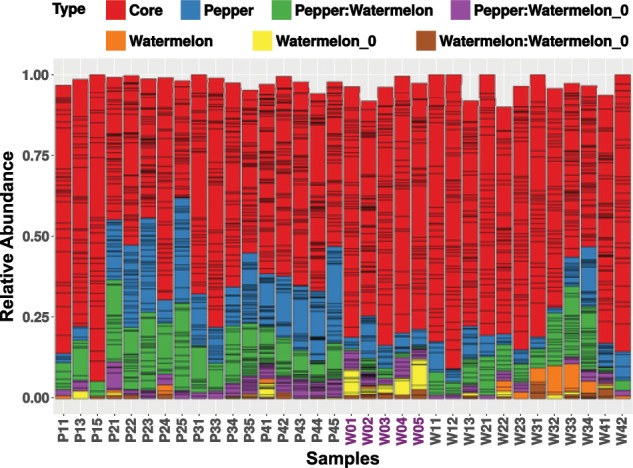
Relative abundance of ZOTUs, collapsed at the genus level (delimited by black lines), and classified as core, pepper field specific, watermelon field specific, starting population specific (Watermelon_0), and genera shared between pairwise combinations according to their presence among *B. tabaci* gut-enriched samples. ZOTUs were classified as core, shared, or specific and were considered part of a certain group only if they were present in at least two samples of each of the subgroups of the main presented biological group. For example, the pepper field-specific main group included four subgroups (generations 1–4). Note that ZOTUs associated with the same group (e.g., pepper field-specific) are coded by the same color (e.g., blue), and may also appear, in a nonconsistent matter, in samples associated with a different biological group. Therefore, although the “blue” labeling appears in all samples, it stands, in each of the non-pepper samples, for different ZOTUs. Summed relative abundance does not reach 1 in some samples as sample-specific ZOTUs were excluded.

In general, gut-associated bacterial communities in *B. tabaci* were mainly composed of three phyla: Proteobacteria (50.4% ± 7.6 SD), Actinobacteria (27.8% ± 7.8 SD), and Firmicutes (15.3% ± 6.8 SD) (Fig. 1). Cyanobacteria (2.5% ± 1.4 SD), Planctomycetes (1.3% ± 2.1 SD), and Acidobacteria (1.2% ± 1.2 SD) phyla were found in low amounts, mainly in gut samples collected from adults feeding on pepper. Pepper samples from the second generation showed a specific increase in the Planctomycetes (5.6% ± 2.1 SD), Chloroflexi (4.1 ± 2.2 SD), and Armanitomodales (2.9% ± 1.1 SD) phyla. Next, we calculated the averaged content of the core, shared, and specific sets of ZOTUs (see definition in “Materials and methods” section) among the three main groups of samples: the starting population (W0), and the two field populations that developed for four generations on pepper (P1–P4) or watermelon (W1–W4) plants (Fig. 2). These analyses produced three main insights: (i) a core set of bacterial taxa, shared by the starting population and the two field populations was identified. It was found to include 104 ZOTUs from five phyla (Actinobacteria, Acidobacteria, Cyanobacteria, Firmicutes, and Proteobacteria), which grouped into 50 genera (Fig. 2 and Fig. S2). This core was not equally distributed (one way ANOVA, *p* value < 0.01) among insects growing on pepper (0.45% ± 0.17 SD core mean) when compared with those growing on watermelon (0.59 ± 0.12 SD core mean). Specifically, the Proteobacteria, Firmicutes, and Actinobacteria phyla had lower representation in insects growing on pepper (one way ANOVA, *p* value < 0.05). Genera with uneven distribution included: *Pseudomonas* and *Enhydrobacter* (Proteobacteria), *Streptococcus* and *Paenibacillus* (Firmicutes), and *Marmoricola* (Actinobacteria). In contrast, genera with even distribution included: *Acinetobacter*, *Pelomonas*, and *Sphingomonas* (Proteobacteria) and *Blastococcus*, *Micrococcus*, and *Nocardioides* (Actinobacteria). (ii) Insects reared under field conditions were found to harbor a large number of ZOTUs that were not present in the starting population. Those reared on pepper had the largest number (145) of unique ZOTUs that grouped into 47 genera, while in those reared on watermelon, 13 unique ZOTUs belonging to three genera were identified. Both field populations shared 55 ZOTUs that grouped into 26 genera. The starting population was found to harbor 24 unique ZOTUs that grouped into nine genera, and shared 38 ZOTUs (12 genera) and six ZOTUs (three genera) with the pepper and watermelon field populations, respectively (Fig. S2). (iii) A nonsignificant trend in diversity indices was detected, as samples from the pepper field population presented, in general, higher diversity indices than both the starting population and the watermelon field population. However, the first generation on pepper presented the lowest diversity (Table S1).

### *B. tabaci’s* gut-associated bacteria β diversity

**Fig. 3:**
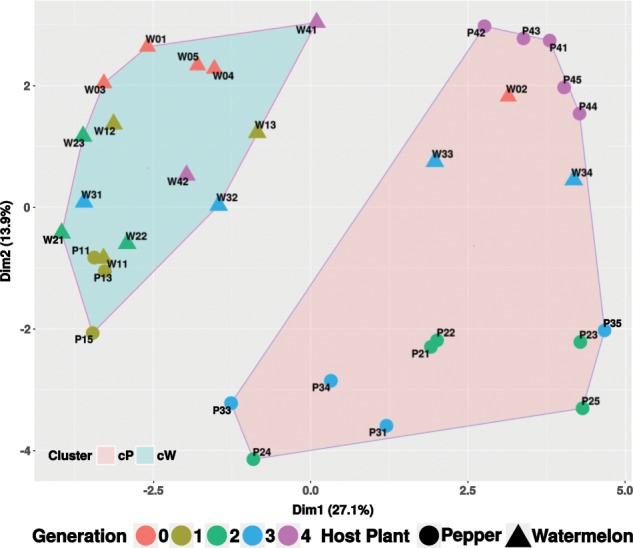
Partitioning around medoids (PAM) coarse level clustering showing the watermelon (cW) and the pepper (cP) enriched clusters of *B. tabaci* gut microbiomes.

Ordination/clustering analysis indicated that host plant and generation time affected the samples grouping, the latter being more a characteristic of the pepper field-population samples (Fig. S3A). The major ZOTUs that drove the separation of the different clusters belonged to the families *Mycobacteriaceae*, *Pseudomonadaceae*, *Moraxellaceae*, *Comamonadaceae*, and *Staphylococcaceae*. Pepper samples from the second, third, and fourth generations showed a characteristic presence of *Mycobacteriaceae* and *Staphylococcaceae*. The first and second generations on watermelon and the first generation on pepper were defined by the presence of *Comamonadaceae* and *Micrococcaceae* (Fig. S3B). Next, we checked for the presence of statistically supported groups at a coarse level, using the majority rule strategy and three clustering algorithms. Two supported clusters were recovered (homogeneity of dispersion or betadisper *p* value > 0.1, permanova *p* value < 0.001, Fig. 3). One cluster was significantly enriched in samples from watermelon, while the other was significantly enriched in samples from pepper. Two exceptions were identified: samples from the first generation on pepper clustered within the watermelon group, and three samples from the watermelon population (W02, W33, and W34) clustered within the pepper group. At higher resolution, four clusters were identified (Fig. S4). While the watermelon samples maintained their clustering singularity, the pepper samples separated into three groups corresponding to generations second to fourth (betadisper *p* value > 0.2, permanova *p* value < 0.001, Fig. S4). When only the generation variable was tested, supported clustering was found in both the watermelon and pepper samples (betadisper *p* value > 0.5, permanova *p* value < 0.001).

At the qualitative level, the second to fourth pepper field generations presented an increase in unique ZOTUs when compared with the starting population, strengthening the generational effect in these samples (Fig. S5A). In contrast, the first generation on pepper and the watermelon field generations, with the exception of two samples in the third generation, presented few, if any, unique ZOTUs and their microbiomes were mainly composed by ZOTUs shared with the starting population (Fig. S5A). At the quantitative level, differentially abundant ZOTUs were assessed. Both watermelon and pepper field populations experienced a significant decrease in bacterial abundance, with the major losses happening in core genera/ZOTUs from the Actinobacteria, Proteobacteria, and Firmicutes phyla, when compared with the starting population (Fig. S5B). At the same time, an increase in several genera/ZOTUs was detected mainly in the second to fourth pepper field generations and the third generation on watermelon: *Craurococcus*, *Chroococcidiopsis*, *Hypomicrobium*, *Methylobacterium*, *Mycobacterium*, *Pseudonocardia*, and *Rhizobium*. Some of these genera/ZOTUs were previously identified as major drivers of the separation between the watermelon and pepper-associated clusters (Fig. 3).

### Host plant effect on *B. tabaci’s* gut-associated bacteria

**Fig. 4:**
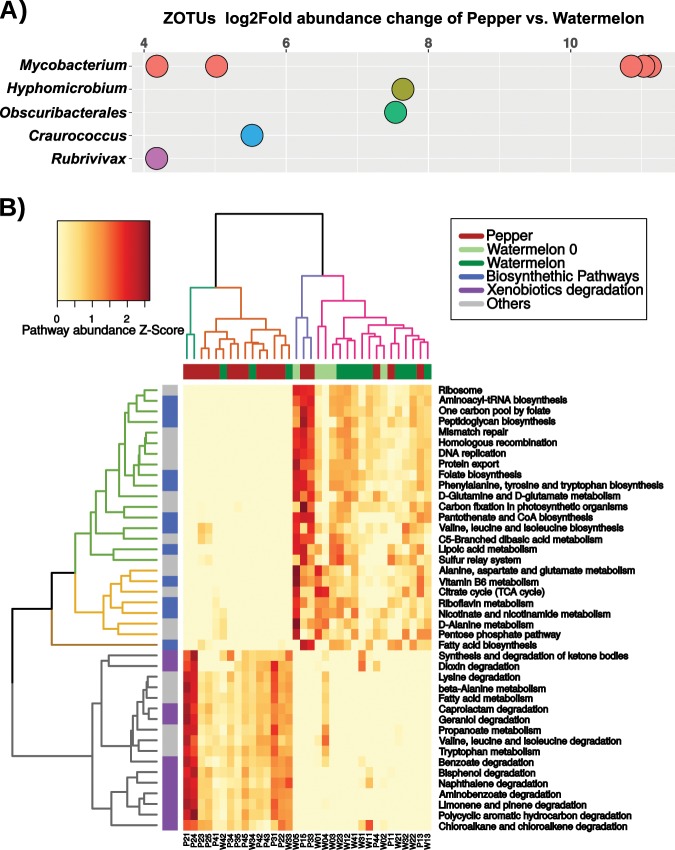
Differentially abundant ZOTUs and metabolic potential of *B. tabaci* gut-associated bacteria. **a** Differentially abundant ZOTUS between pepper and watermelon. ZOTUs were independently detected by both the DESeq2 and random forest classifiers. Positive log2Fold values indicate greater abundance in the pepper group compared with the watermelon group. Dots with the same color represent different ZOTUs within the same genus. **b** The inferred metabolic potential of gut-associated bacterial communities of *B. tabaci* growing on pepper and watermelon plants. Only LEfSe discriminative pathways are shown. The dendrogram with turquoise and orange columns highlights the pepper cluster, while the purple and pink columns dendrogram highlights the watermelon cluster. Similarly, the dendrogram with gray colored rows highlights pathways associated with the pepper cluster, while the brown, yellow, and green rows dendrogram highlights pathways associated with the watermelon cluster. *Z*-Score (standardized score) for each pathway was calculated by subtracting the mean of all samples from the value of each specific sample, and dividing the obtained difference by the standard deviation of the mean.

To identify specific ZOTUs that showed differential abundance in the watermelon and pepper clusters, we took into account only ZOTUs that were independently detected by both the DESeq2 and random forest classifier. Nine ZOTUs were found to be, at least, 16 times more abundant in the pepper cluster than in the watermelon cluster (Fig. 4a). From them, six were classified as part of the *Mycobacterium* genus (Actinobacteria phylum), representing ~5–10% of the total diversity of the gut-associated bacteria in the second, third, and fourth generations on pepper. Phylogenetic analysis indicated that five of the *Mycobacterium* ZOTUs belong to one clade and cluster together with *M. fortuitum*, while the sixth, ZOTU60, grouped in a different clade together with *M. peregrinum* (Fig. S6). The rest of differentially abundant ZOTUs were assigned to the genera *Craurococcus* and *Hyphomicrobium* (Alphaproteobacteria), *Obscuribacterales* (Cyanobacteria), and *Rubrivivax* (Betaproteobacteria) (Fig. 4a). To further explore the observed differences in the gut-associated bacterial communities, we inferred their metabolic potential and the relative abundance of the different metabolic pathways using PICRUST/HUManN. Then, we used LEfSe to predict metabolic pathways that may vary between the pepper and watermelon samples (discriminative features with LDA > 2.5 and 1000 bootstraps, Fig. 4b). Insects developing on pepper harbored microbiomes with a significant enrichment in various xenobiotics degradation pathways (e.g., bisphenol, limonene, and geraniol), while insects developing on watermelon harbored microbiomes that were enriched in biosynthetic pathways (e.g., vitamins, amino acids, and precursors) (Fig. 4b). These pathways successfully clustered the samples according to their host plant, with few exceptions: samples of the first generation on pepper, P33 and P44 that clustered with the watermelon samples, and watermelon samples W33, W34, and W42 that clustered with the pepper samples.

### Survival of *B. tabaci* on watermelon and pepper plants along four generations

**Fig. 5:**
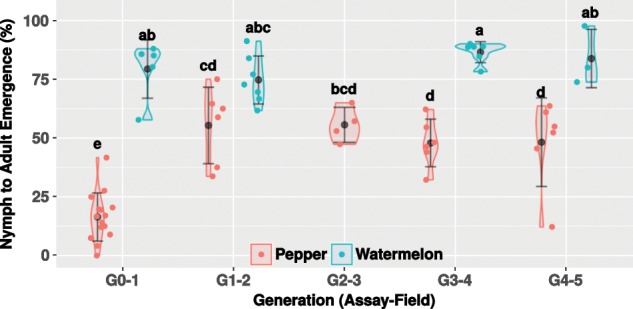
Performance (egg-to-adult survival) of the field populations on their respective host plant, under controlled environment, along four generations. Data from two (G1–2 to G4–5) and five (G0–1 as performance baseline, recorded each generation) independent experiments are represented as violin plots, with each dot representing whiteflies’ performance on single leaves (three leaves in average per plant). Average survival and standard deviation are denoted by filled black dots and whiskers, respectively. Letter/s above each violin plot indicate/s the significance of the differences after conducting a Tukey’s HSD test. The G0–1 assay, which is the first time the starting population was transferred to the field plots, correlated with the first generation that have developed in the field (P1 or W1). Similarly, the G1–2 assay correlated with the second generation that have developed in the field (P2 or W2).

Every generation, adult insects from the watermelon and pepper field populations were collected and transferred to a controlled greenhouse for testing their progeny egg-to-adult survival on their respective host plant (Fig. 5). Three significant patterns (two-way ANOVAs, *p* value < 0.05) were observed (Fig. 5). First, the insects’ survival on watermelon was stable over the generations (80.5% ± 10.35 SD). Second, the performance of the watermelon field population on watermelon was always higher than the performance of the pepper field population on pepper. Third, after displaying high mortality levels on pepper during the first generation (G0–1, 16.44% ± 10.25 SD), the survival increased and kept stable over subsequent generations (51.43% ± 13.92 SD) (Fig. 5).

## Discussion

Culture-independent analyses have previously suggested that sternorrhynchan insects present low levels and variability of gut-associated bacterial communities [10, 12, 13]. In contrast, our strategy of combining endosymbiotic sequences depletion by bDPO and deep sequencing of gut RNA samples, brings clear evidence that *B. tabaci* holds a significant, dynamic, and active gut-associated bacterial community. The whitefly’s gut was dominated by the phyla Proteobacteria, Actinobacteria, and Firmicutes, resembling previously reported insect’ gut microbiomes, which are mainly dominated by Proteobacteria (57–65%) and Firmicutes (7–22%), but with variable amounts of Actinobacteria (5–8%) and Bacteroidetes (6–15%) [8–10]. The occurrence in the gut microbiome of the genera reported here (e.g., *Acinetobacter*, *Bacillus*, *Micrococcus*, *Moraxella*, *Mycobacterium*, *Pseudomonas*, *Staphylococcus*, and *Sphingomonas*), including those classified as part of the core set, is in agreement with previous culture-dependent and -independent analyses of *B. tabaci* [20, 21, 29, 30] and other sternorrhyncha species [31, 32] microbiota (Fig. S7). Taken together, these findings raise the possibility that some environmental bacteria are capable of establishing recurrent associations with sternorrhyncha insects, including whiteflies, and can be considered as consistent gut residents.

The next question that arises is how these gut bacteria are acquired? As already indicated, most of the aforementioned gut-associated microbial communities of *B. tabaci* were classified as environmental or plant-associated bacteria, suggesting their acquisition during the landing, evaluation, and feeding steps on the host plant [11]. Furthermore, it has been previously shown that whiteflies can acquire bacteria by feeding on artificial diets containing isolated gut-associated bacteria [21]. Similarly, acquisition from plant tissues can occur during the stylet movement through the apoplast, toward the phloem sieve element [21, 33, 34]. In addition, whiteflies and aphids occasionally feed on the xylem sap [35, 36], a behavior that has been linked with the maintenance of water balance or with the need to counterbalance the high osmotic pressure of the phloem [36, 37]. Moreover, when encountering a less-suitable host plant, whiteflies seem to feed for longer periods on the xylem, and reduce their phloem ingestion [38]. The accumulated xylem feeding periods can lead to the acquisition of a more diverse bacterial community than that gained when feeding mainly on the phloem [39, 40]. In this respect, it is important to note that some matters that were not addressed here require investigation in future studies. These include mainly the comparison between the host-plant microbiome and the insect microbiome. This can lead, for example, to the identification of bacteria capable of colonizing both plants and insects. These bacteria, which may have different roles in the two “host environments”, are likely to be of special interest both from the entomological and botanical point of views and may also have biotechnological potential. Moreover, it can lead to the identification of certain bacteria that can be acquired by the insect only from a specific host plant. This can expand our understanding of how some environmental bacteria are capable of establishing recurrent associations with sternorrhyncha insects. Also, it will help to determine the mode of transmission of these bacteria when the insect switches to alternative plant hosts.

Although specific behaviors that can ensure parent-to-offspring transmission of gut-associated bacteria have not been recorded in sternorrhynchan insects, putative mechanisms such as honeydew and co-feeding may serve as bacteria inocula [41]. Honeydew is a sugar-rich medium that can support the growth of a starting bacterial inoculum, allowing the resident gut-associated bacteria to colonize the leaf surface (epiphyte). In addition, some insect endosymbionts and pathogenic endophytes (e.g. *Liberibacter*) can be transferred to and from plants. Hence, it is possible that some environmental bacteria can be transmitted from parents to offspring by feeding on the same host plants. For example, the deposition on the leaf surface of a pathogenic *Pseudomonas syringae* strain, capable of colonizing the pea aphid gut, together with honeydew, enabled its transmission to the aphid offspring [33]. In the wheat aphid, nymphs co-feeding with their mother presented similar microbiomes while isolated nymphs presented much lower bacterial loads and diversity [42]. Other studies also brought evidence for plant-mediated transmission of gut-associated bacteria in aphids (*Serratia symbiotica*) [43] and endosymbiotic bacteria in whiteflies (*Rickettsia*) [44].

**Fig. 6:**
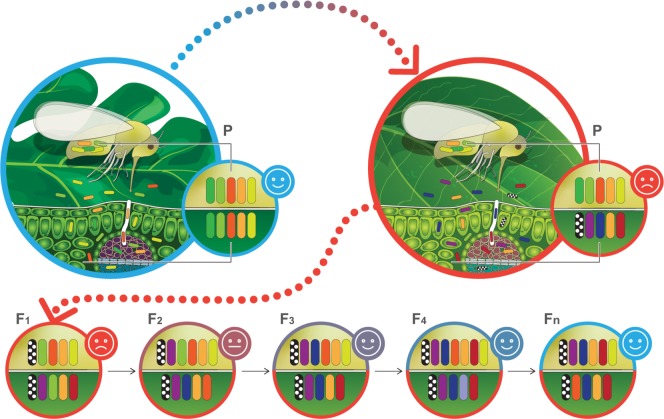
A hypothetical scenario for sap-feeding insect gut-associated bacteria acquisition during switching from a suitable (blue encircled leaf) to a less-suitable (red encircled leaf) host plant. Each generation (bottom circles), the insect (upper yellow halve) acquires new bacteria (colored bacilli) from the host plant (bottom green halve) or by indirect parent-to-offspring transmission mechanisms (represented by the same bacilli over generations), which modify its gut microbiota community. This process can last until a community providing a local optimal performance is assembled. Smileys represent insects’ performance on the host plant, ranging from low (red) to high (blue) performance. The dynamics of both plant and insect microbiomes is represented by gain/loss of bacilli at each generation.

Our data indicated that the acquisition of gut-associated bacteria by *B. tabaci* is strongly affected by the identity of the plant host, as already proposed in other insect systems [8–10]. *B. tabaci* populations that developed on pepper for more than one generation harbored a higher microbial diversity than the populations developing on watermelon, and presented a large fraction of pepper-specific bacteria, likely in the expense of a quantitative reduction of core bacteria. As we observed not only a significant change in the gut-associated bacteria between the first generation and the subsequent ones on pepper, but also among the successive generations, the following scenario can be suggested (Fig. 6): the transfer from laboratory to field-like conditions produces a disruption of the initial gut-associated bacterial communities, as both the insect host and its gut-associated bacteria are facing a new environment. Subsequently, the insect and its gut-associated bacteria go through a multigenerational adaptation period to the new host plant and/or environment which continuously modifies the assemblage of the bacterial communities (e.g., enrichment/acquisition of pepper-specific bacteria) [45], largely due to direct effects of available nutrients and/or secondary metabolites. These include, among others, flavonoids, phenols, and capsaicinoids [46]. Previous studies have indicated cases in which gut microbiota were shown to be capable of detoxifying poisonous plant compounds including flavonoids, alkaloids, terpenes, and isothiocyanates [47–49]. As long as the selective pressure is maintained (e.g., nutritional limitations and/or the presence of phytotoxins in the diet), the microbiome is predicted to move toward a local performance optimum. This means that new bacterial populations can be acquired and previous ones can be lost due to competition, lack of transmission capabilities, or random events. Under this scenario, stability is predicted to be more likely achieved at the community and/or metabolic functional levels rather than the taxonomic level [50, 51].

In our study, a significant enrichment of the microbiome with *Mycobacterium* ZOTUs was observed in advanced generations on pepper, as well as a predicted higher metabolic potential, enriched in xenobiotics and secondary metabolites degradative pathways, which correlated with a significant increase in survival on pepper plants from the second generation onward. The genus *Mycobacterium* is divided into fast- and slow-growing bacteria. Fast-growing species are mainly considered environmental-related bacteria, although some species can be opportunistic parasites. In contrast, slow-growing *Mycobacterium* are usually related to a parasitic lifestyle [52]. Several fast-growing *Mycobacterium* species have been described as plant-associated epi/endophytes [40], while others were reported as gut-associated bacteria of Lepidoptera and aphid species [23, 53]. *Mycobacterium* are known to produce biofilms, allowing them to adhere to different surfaces, but also to overcome unfavorable abiotic and biotic environments [54]. These capabilities ensure both colonization and survival in the adverse conditions present in the insects’ gut following acquisition from the plant/environment [6]. In addition, some *Mycobacteriums* show incredible bioremediation capabilities, by being able to degrade complex compounds, such as isoprenoids or alkanes [55]. It is therefore possible that some endophitic *Mycobacterium* might have the ability to counteract, by degradation and/or assimilation, different toxic secondary metabolites present in pepper plants, for ensuring their own survival [56].

Although technical limitations currently precludes it’s testing (it is impossible to infect or cure a population with a specific environmental bacterium), the following possibility is suggested: in the insect’s gut, the presence/acquisition of bacteria with predicted metabolic capability/s to degrade/assimilate secondary toxic metabolites has the potential of providing a significant benefit to the host [57, 58]. As long as the selective pressure is maintained, the offspring seem to be capable of acquiring their parents’ microbiome, either by co-feeding or indirectly from the honeydew, allowing the overgenerational maintenance of putative beneficial bacteria in the local population (Fig. 6). This might synergize or complement insect-specific short adaptation mechanisms to less-suitable host plants that are well documented and largely involve metabolic adaptation by the overexpression of genes coding for detoxification enzymes and xenobiotic transporters [2, 59]. Further analyses are required in order to determine the exact contribution of each component to the ability of the population to adapt relatively quickly (second to third generations) to less-suitable host plants.

## Supplementary information

Supplementary Information

## Data Availability

RAW sequences from the field experiment have been deposited in the Sequence Read Archive under the BioProject accession number PRJNA525688. All RAW sequences (including the trial experiment—see Supplementary Information), relevant code, metadata, and statistical analysis can be found at 10.6084/m9.figshare.5955880.
