# Supplementary material for: Inside out: microbiota dynamics during host-plant adaptation of whiteflies
Source: ISME J. 2020 Jan 2;14(3):847–56. doi: 10.1038/s41396-019-0576-8 (PMC7031279; doi:10.1038/s41396-019-0576-8)
Supplement: Supplementary file 1 — Supplementary Information [file 41396_2019_576_MOESM1_ESM.pdf]

Inside out: microbiota dynamics during host-plant adaptation of whiteflies
Supplementary Information

Diego Santos-Garcia[1], Natividad Mestre-Rincon[1], Einat Zchori-Fein[2], Shai Morin[1]

[1]Department of Entomology, The Hebrew University of Jerusalem, Rehovot, Israel

[2]Department of Entomology, Newe-Ya'ar Research Center, ARO, Ramat-Yishai, Israel

Corresponding author: Diego Santos-Garcia, Department of Entomology, The Hebrew University of Jerusalem, P.O.Box 12, Rehovot 7610001, Israel.

Phone: 972 89489365

E-mail: diego.santos@mail.huji.ac.il, diego.santos.garcia@protonmail.com

Extended Materials and Methods

Insects, Plants and field experiment

On March 2015, few thousands *Bemisia tabaci* adults were collected from an insecticides-free watermelon field (*Citrullus lanatus* var. *lanatus* cv. 'Malali') near Menahamiya in the Jordan valley region of Israel (GPS 32.6164, 35.549604). Whiteflies were transferred to rearing chambers ($27{\pm}2°C$, 16h:8h L:D, 50-55% humidity), and the population was boosted for few generations using the same watermelon cultivar. The collected population was identified as the Middle East Asia Minor 1 (MEAM1) species of the *B. tabaci* species complex based on the sequence of the mitochondrial cytochrome C oxidase subunit I gene (*mtCOI*, sequences are available at 10.6084/m9.figshare.5955880) [1]. The MEAM1 species of *B. tabaci* can be considered as a "true" generalist, as it is able to feed on crops and weeds from 49 botanical families according to an exhaustive literature survey [2]. The population was found to harbor the obligatory endosymbiont *Portiera* and the facultative endosymbionts *Hamiltonella* and *Rickettsia* (Table S2). *Portiera* and *Hamiltonella* endosymbionts are restricted to the bacteriocytes (specialized insect cells). In contrast, *Rickettsia* can be found infecting cells of different tissues, including the gut epithelium [3, 4]. However, at least in MEAM1, *Rickettsia* was never found to be present in the gut lumen [5]. Therefore, we considered all three endosymbionts as non-gut-associated bacteria.

From late July - December 2015, the season characterized by abundant populations of *B. tabaci* in Eastern Mediterranean climate [6], a field-like experiment (Fig. S8) was conducted at the Hebrew University Experimental Farm in Rehovot, Israel (GPS 31.904405, 34.797097). The boosted *B. tabaci* colony was used as the starting population. Two host plants (diets) were used throughout the experiment: pepper (*Capsicum annuum* var. *annuum* cv. 'Hungarian hot wax') as the "treatment" diet, and Malali watermelon as the "control" diet. While watermelon can be considered as a suitable host for *B. tabaci* [7, 8, 9], several pepper cultivars were reported to be less-suitable host plants for the MEAM1 species [10, 11, 12, 13, 14, 15].

Two neighboring plots, covered by anti-birds nets, were planted along their inner edges, with one of the selected host plants. In the middle of the plot, an experimental platform was placed (Fig. S8). Single watermelon or pepper plants were transferred from the greenhouse and planted in 10 liters buckets filled with *in-site* soil. Plants were placed inside BugDorm-2120F insect

rearing tents (MegaView Science) standing on the experimental platform (five plants/tents per plot). Three days later, *B. tabaci* adults were introduced into each tent, and the sub-population was allowed to complete a generation (from egg laying to adult emergence). The newly-emerged adults were collected. The tents were cleaned (soap and ethanol 70%) and new plants, with *in-site* soil, were placed in each tent. Then, the newly-emerged adults were released in order to produce the next generation. Tents were labeled and kept as isolated sub-populations (no mixing of adults between tents along the generations).

The tents used, had two lateral polyester meshes (160 μm pore) that allowed biotic (mainly bacteria, fungi and small arthropods but not *B. tabaci* adults) and abiotic (mainly wind, dew and dust) factors to pass freely from the surrounding environment (outside the netted plot and planted plants along the plot's inner edges) to the inner plants within the experimental tents. The surrounding fields were not treated with insecticides or herbicides for at least four years. Each generation, the experiment was monitored every two days until day 15 and daily thereafter. Spider mites infestations were biologically controlled using the specialist predatory mite *Phytoseiulus persimilis*. Mealybugs were manually removed upon detection.

Performance assays

Each generation, newly-emerged adults were transferred from the field experiment to a controlled greenhouse ($25\pm3°C$, 16h:8h L:D) for conducting performance assays. Five adult couples (mixed from the five tents) were placed in each clip-cage that was attached to a leaf, and allowed to oviposit for a period of 24 h on new watermelon or pepper plants (according to their field hostplant). Two plants, with three clip-cages (one per leaf), were set for each treatment. After the oviposition period, the number of eggs per leaf was recorded, and their development was monitored daily until all adults emerged, or 35 days passed from the first emergence (a time point in which all non-emerged individuals were considered dead). As a baseline, the performance of the starting population on pepper and watermelon plants was recorded each generation. Statistical analyses were conducted in R and are available at 10.6084/m9.figshare.5955880. Damaged leaves or leaves harboring less than five eggs were discarded. Shapiro's and Levene's tests were used to check for data normality and homocedasticity before running two-way ANOVA tests ("host

plant" and "generation" as the main effects).

Gut-enriched RNA samples, total RNA extraction and first strand cDNA synthesis

Collected adult females (24/48 hours after emergence) were left unfed for two hours in order to allow them to evacuate their gut content [16], and were then surface sterilized under a UV-C lamp for 30 min. The UV-C treatment was preferred over the ethanol/bleach standard one, because the dehydration produced by the ethanol interferes with successful gut dissection.

For each RNA sample, 12 females per tent were dissected and their guts pooled (Fig. S8). Dissections were performed in cold 1X Phosphate Buffered Saline (autoclaved and filtered through 0.2 μm syringe filters), using sterilized dissecting tools under a stereo microscope placed inside a level one biosafety cabinet. Guts were transferred directly to clean tubes containing lysis buffer (NucleoSpin RNA XS; Macherey-Nagel) and 0.1 mm zirconia beads (on ice), and homogenized using a bead-beater (Minilys, Bertin Technologies) at maximum speed for 30 sec. The next steps for total RNA extractions, were performed following the instructions of the NucleoSpin RNA XS manufacturer (Macherey-Nagel).

The RNA was reverse-transcribed to cDNA using a two steps protocol. First, 8 μl of RNA, 1 μl of random hexamers and 3.5 μl of RNAse free water were mixed and left for 5 min. at 65°C. Then, 4 μl of 5X Reaction Buffer, 0.5 μl of RiboLock RNase Inhibitor (Thermo Fisher), 2μl of dNTPs [10nM] and 1μl of RevertAid H Minus Reverse Transcriptase (Thermo Fisher) were added (20 μ final volume). All steps were performed inside a UVC/T-AR DNA/RNA UV-cleaner cabinet with 30 min. of UV-C exposure between each step. The reverse-transcription PCR included 10 min. at 25°C, 60 min. at 42°C and 10 min. at 70°C. cDNA was kept at -80°C until usage.

MiSeq trial and Dual Priming Oligonucleotide design

A sequencing trial of six gut-enriched samples, was conducted using the 319F (V3) and 806R (V4) 16S rRNA universal primers (Table S3). One single master mix was set up as follows: 80 μl of KAPA2G Robust HotStart ReadyMix polymerase; 0.8 μl of each of the 319F and 806R universal primers (10 mM each), 14.4 μl of water (RO PW Sterile Water, Biological Industries). Final

reactions contained 8 μl of cDNA and 12 μl of the master mix, giving a final concentration of 0.05 mM to each of the universal primers. The PCR conditions were as follows: 95°C for 5 min., [95s for 30 sec., 55°C for 15 sec., 68°C for 30 sec.]*30, and 68°C for 7 min. All reactions used reagents/enzymes from the same batch. PCRs were prepared inside a UVC/T-AR DNA/RNA UV-cleaner cabinet. PicoGreen dsDNA HS kit was used to measure the concentration of the PCR products. Amplicons were sent to the DNA Services Facility at the University of Illinois (Chicago). The samples were multi-tagged by a second PCR, and sequenced using a MiSeq machine with the V2 chemistry (2x250 base paired reads).

All sequences obtained in the trial were mainly classified as *Portiera* and *Hamiltonella* (Fig. S9). Therefore, a PCR-blocking strategy was developed using the dual priming oligonucleotides (DPOs) strategy. DPOs are two separate primers linked by a deoxyinosine bridge [17], which allows both regions to anneal independently, getting highly specific primers to anneal at low temperatures. Blocking DPOs (bDPOs) end in a C3 spacer at the 3'-end, which blocks PCR elongation (Fig. S10). The obtained sequences were used to design specific bDPOs, which overlapped with the 515R (V3) universal primer 3'-end (one for each endosymbiont, Table S4). ProbeCheck [18] was used to test (4 mismatches allowed) the specificity of the bDPOs selected. A competition assay was conducted (10-300 times more bDPOs than 515R), using the same PCR conditions as described below, to establish an acceptable blocking ratio. Blocking ratio was calculated by cloning and Sanger sequencing of ten clones from each competition assay. All scripts and RAW sequences of the reported MiSeq trial are available at 10.6084/m9.figshare.5955880.

MiSeq sequencing of field experiment

One single master mix was set up as follows: 500 μl of KAPA2G Robust HotStart ReadyMix polymerase; 0.5 μl of each of the 27F (V1) and 515R (V3) universal primers (100 mM each, Table S3); 83 μl of *Portiera*, 8 μl of *Rickettsia*, and 8 μl of *Hamiltonella* bDPOs (100 mM each) [17, 19]. Final reactions contained 8 μl of cDNA (33 ng per PCR in average) and 12 μl of the master mix, with a final concentration of 8.3 mM for the *Portiera* bDPO, 0.8 mM for both the *Rickettsia* and *Hamiltonella* bDPOs, and 0.05 mM for each of the universal primers. The PCR conditions were: 95°C for 5 min., [95°C for 30 sec., 55°C for 15 sec., 68°C for 30 sec.]*30, and

68°C for 7 min. All reactions used reagents/enzymes from the same batch. PCRs were prepared inside a UVC/T-AR DNA/RNA UV-cleaner cabinet. PicoGreen dsDNA HS kit was used to measure the concentration of the PCR products.

The final array of PCR samples for sequencing included: 40 gut-enriched samples, a mock community sample (ZymoBIOMICS Microbial Community Standard, Zymo Research) to assess the quality of the RNA extraction method, a negative control sample (without cDNA and processed in parallel) to control for potential environmental/kits/enzymes contamination, and a technical PCR positive control (whole-body homogenate of genomic DNA). PCR products were loaded on a DNAstable 96-well plate (Merck), following the manufacturer instructions (overnight dehydration inside a level 1 biosafety cabinet), and sent for sequencing to the DNA Services Facility at the University of Illinois (Chicago). Universal primers with adapters were used to perform a second PCR (eight cycles) and add the Illumina barcoding primers. Amplicon sequencing was performed by a MiSeq machine employing the V3 chemistry (2x300 base paired reads).

Data pre-processing and clustering

Briefly, RAW reads were processed with cutadapt [20] to remove remaining primers/linkers. Cleaned sequences were piped into the following USEARCH algorithms [21]: *fastq_mergepairs* (paired-reads assembly and filtering), filter_phix (PhiX viral genome removal), *fastq_filter* (fastq quality filtering with maximum expected error threshold set to 2) and *fastx_unique*s (de-replication step). Clustering was performed with USEARCH algorithm *cluster_otus* (minimum filtered reads to build a cluster set to 4) for generating 97% identity clusters, or OTUs (Operational Taxonomic Units), and *unoise3* (minimum abundance set to 10) to compute *denoised* amplicons or zero-radius OTUs (ZOTUs), which are groups of nucleotide sequences with a similarity higher or equal to 99%. Taxonomic classification of OTUs/ZOTUs was performed with mothur's *classify.seqs* command [22] and the SILVA rRNA database Release 128 [23]. Further analyses were only performed on the ZOTUS. A custom bash wrapper was generated (available at 10.6084/m9.figshare.5955880) for data pre-processing, clustering, and taxonomic classification.

Microbiome analysis

Data pre-processing steps included the discarding of: reads obtained from endosymbiotic bacteria, phyla that were not found to be present in at least three samples, ZOTUs with less than three reads or not present in at least two samples, and possible PCR/Kit contaminants (negative control). For the last step, a simple rule was applied: every ZOTU with abundance in the negative control equal or greater than the lower 99% confidence interval of the gut-derived samples, was discarded. Finally, only libraries maintaining more than 2000 reads were kept.

Alpha diversity (Chao1, Shannon, Simpson indexes) and sampling effort curves were calculated with non-transformed samples. In all subsequent analyses, a variance stabilization normalization step was applied to correct for differences in library sizes [24]. Core, shared and specific microbiomes (Euler diagrams) were computed by averaging the abundance of the corresponding phylum/genus/ZOTU and discarding any phylum/genus/ZOTU that did not appear in at least two samples from the same experimental group under analysis (host-plant and/or generation). Beta diversity was explored using normalized libraries, Bray-Curtis distances, and ordination analysis: Principal Components Analysis (PCA), Principal Coordinates Analysis (PCoA), Nonmetric Multidimensional Scaling (NMDS), and Canonical Correspondence Analysis (CCA). Three different clustering methods were applied to Bray-Curtis distances: hierarchical, partition around medoids (PAM) and Markov Cluster Algorithm (MCL). Significance of the clustering results was checked using a PERMANOVA (adonis, inter-group variance) and a beta-dispersion test (intra-group variance). DESeq2 analysis and random forest classifier were used to check for differentially abundant (DA) ZOTUs among enriched clusters or biological groups. Shapiro's and Levene's tests were used to check for data normality and homocedasticity, respectively, before running the ANOVA tests.

Generated ZOTUs were matched to the GreenGenes database IDs (8.15.13 release) using USEARCH *closed_ref* command (97% identity) [21]. The microbiomes metabolic potential were predicted based on the recovered GreenGenes IDs with PICRUSt [25]. Counts were normalized by 16S rRNA gene copy numbers with PICRUSt and the counts table was further summarized with HUManN [26]. LEfSe was used to screen for significant differences in the inferred metabolic pathways between groups of samples [27].

Statistical analyses were performed mainly using the phyloseq, factoextra, vegan, DESeq2 and randomForest packages. All relevant code, metadata and RAW sequences can be found at 10.6084/m9.figshare.5955880.

Phylogenetic inference

16S rRNA gene sequences were extracted from all *Mycobacterium* genomes available in the RefSeq database, discarding those smaller than 1Kb, and dereplicated with CD-HIT-EST. ZOTUs classified as *Mycobacterium* and the *16S* rRNA gene sequences were aligned with mafft (–maxiterate 1000 –localpair) [28] and Gblocks (-t=d -b5=h) [29]. A Maximum likelihood phylogenetic tree was inferred with IQ-TREE (-m MFP -bb 1000 -nstep 500 -alrt 5000) [30]

Supplementary Figures

A)

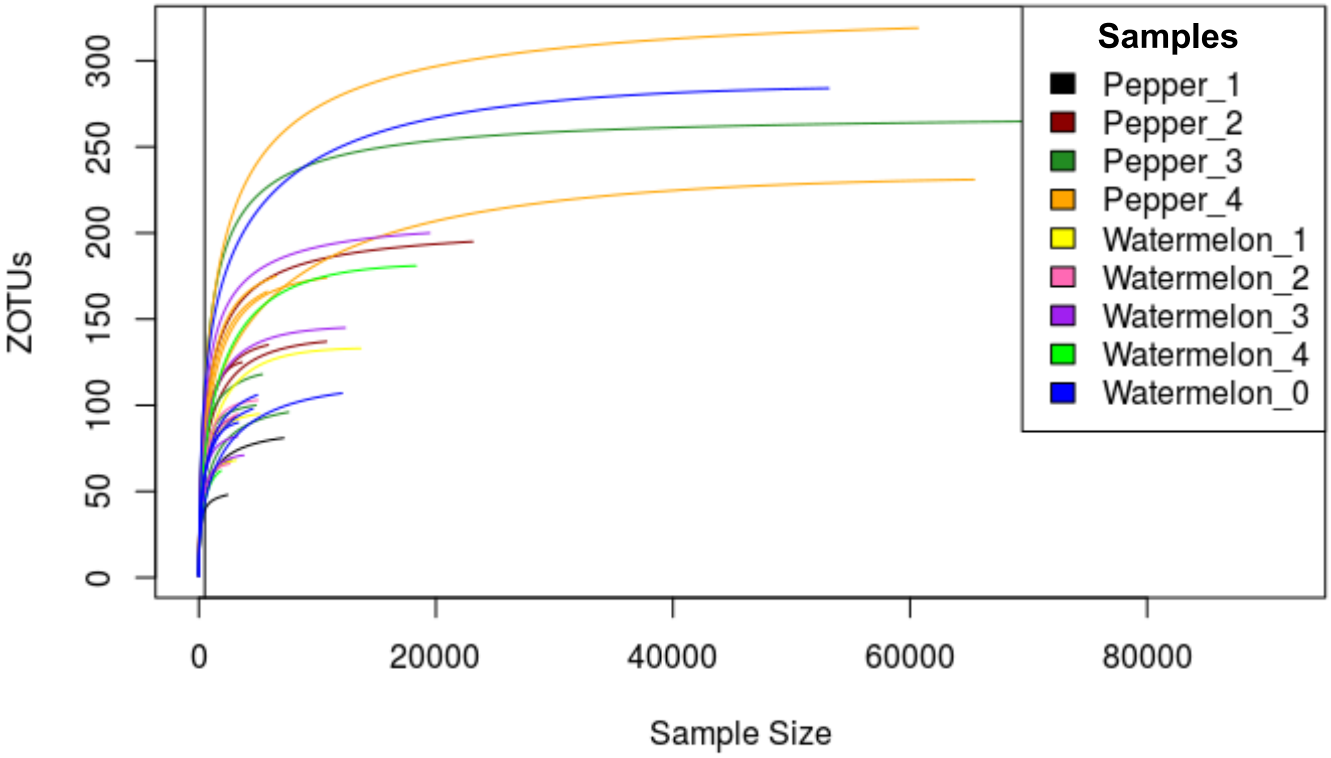

B)

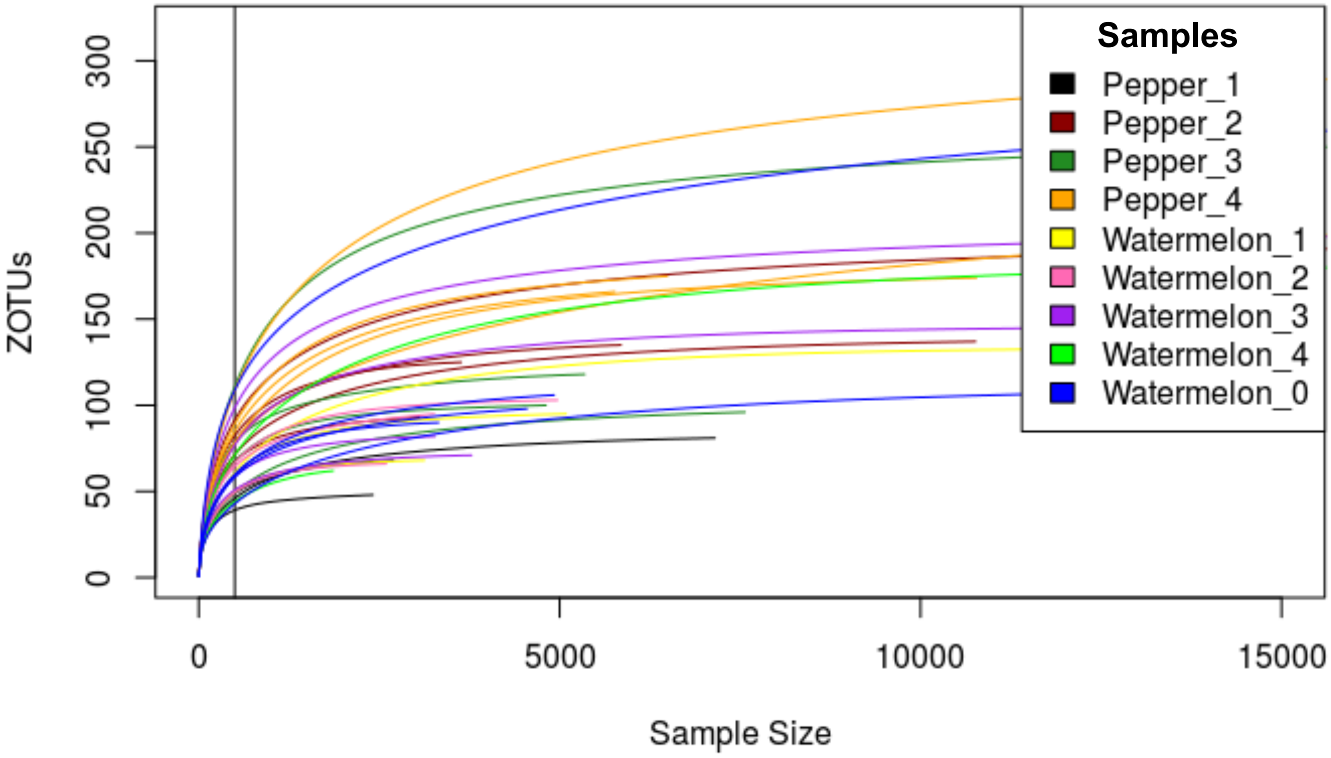

Figure S1: Sampling effort (rarefaction curves, 500 reads sampling step) of each gut enriched sample. Endosymbionts (*Portiera*, *Hamiltonella*, and *Rickettsia*) and possible kit/environment/PCR contaminations were discarded before calculation. **A)** Rarefaction curves with a complete X axis, corresponding to the sample with the largest library size. **B**) Rarefaction curves with a truncated X axis to better visualize small library size samples.

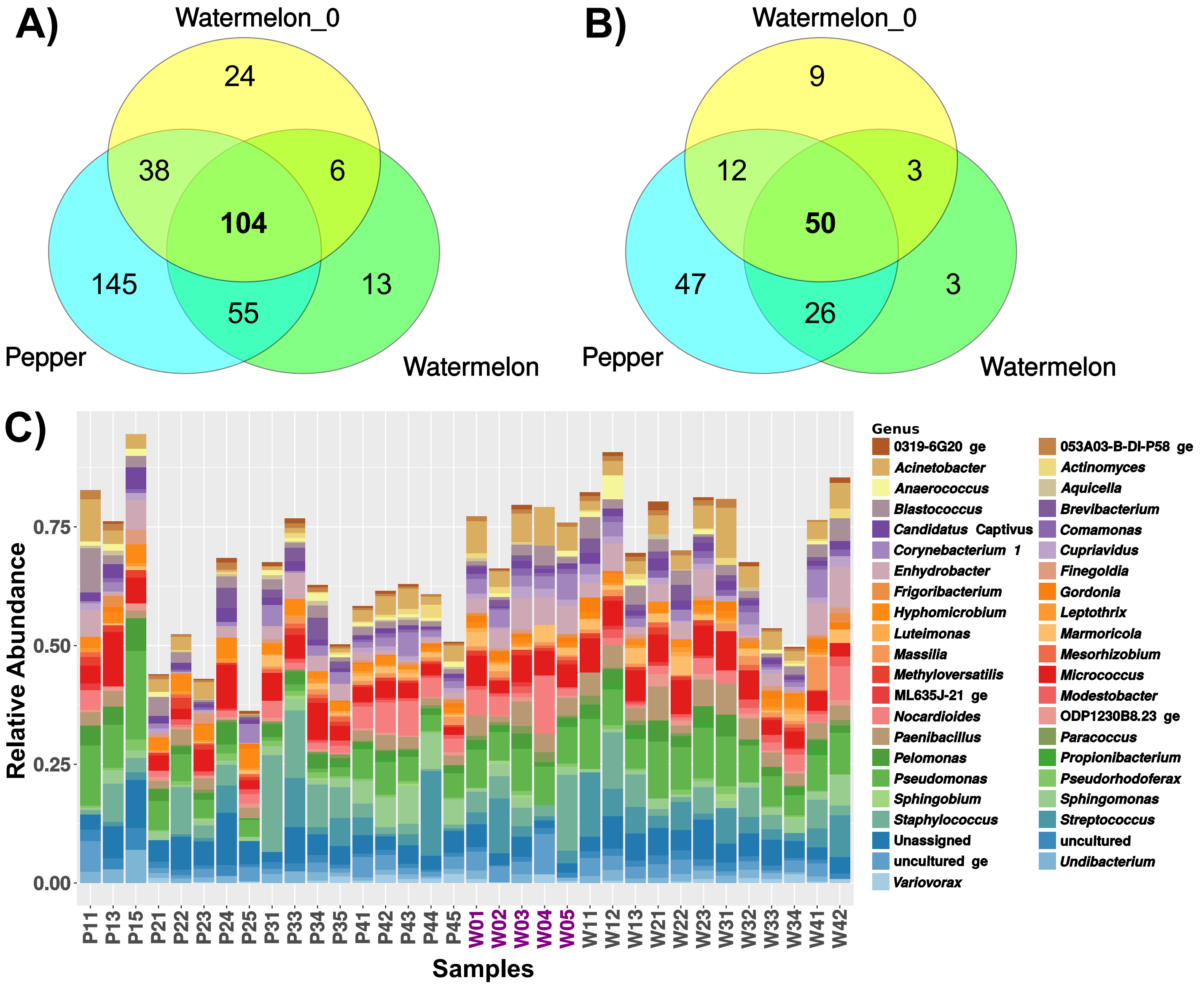

Figure S2: Distribution of ZOTUs according to their classification as core, shared, and unique. Euler diagrams showing the averaged number of **A)** ZOTUs and **B)** genera composing each category. **C)** Relative abundance of the genera composing the core ZOTUs. Seven ZOTUs belonging to different families/classes, were not assigned to a genus, and were collapsed as unassigned. For this reason, only 43 genera instead 50 are displayed. Summed relative abundance does not reach 1 as shared and unique ZOTUs are not displayed. The starting population samples (W0) are highlighted in purple.

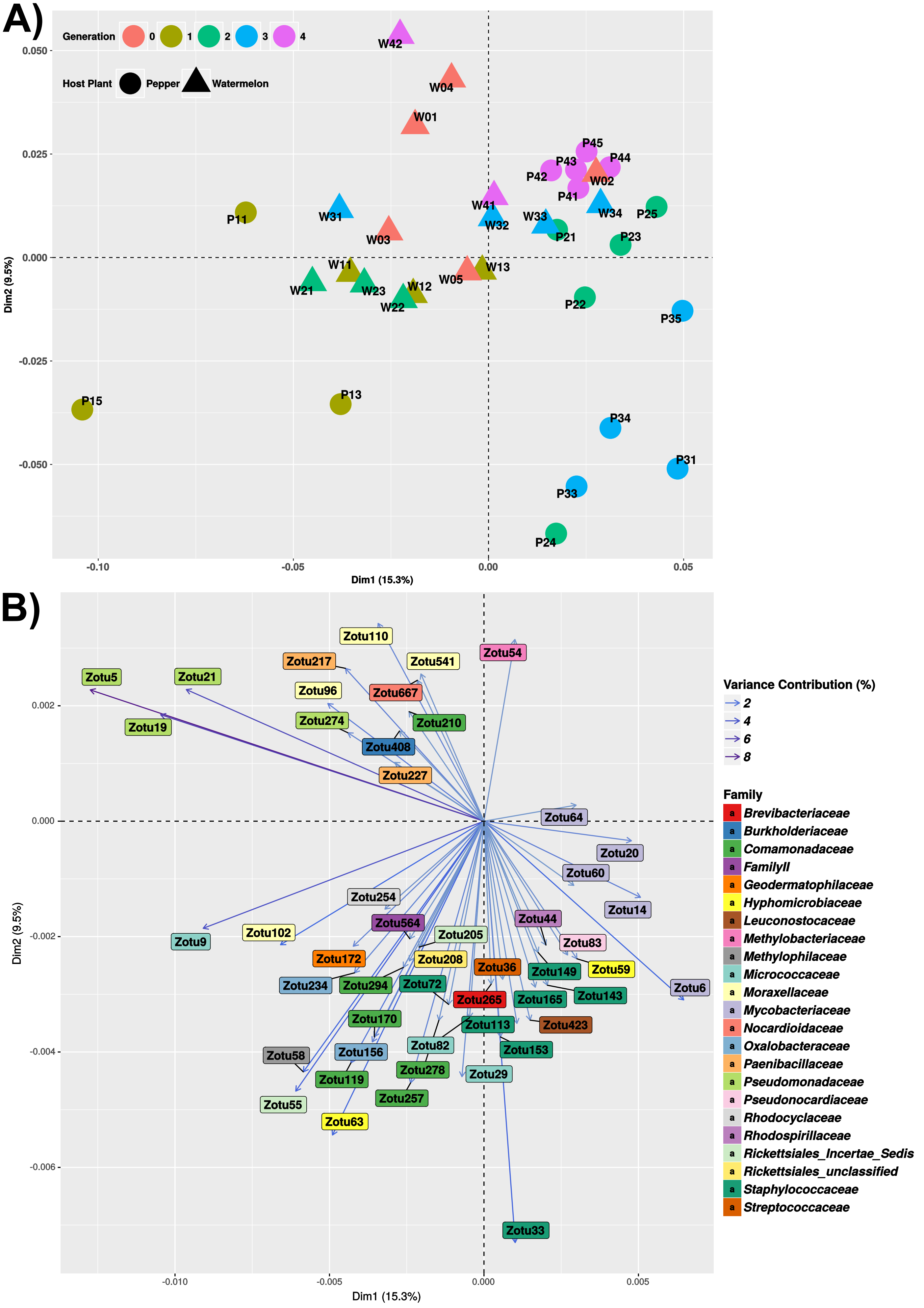

Figure S3: Samples beta diversity. **A**) Principal component analysis (PCA). The two first components explain 24.8% of the variance. **B)** ZOTUs variance contribution. Only the 50 most important ZOTUs are shown.

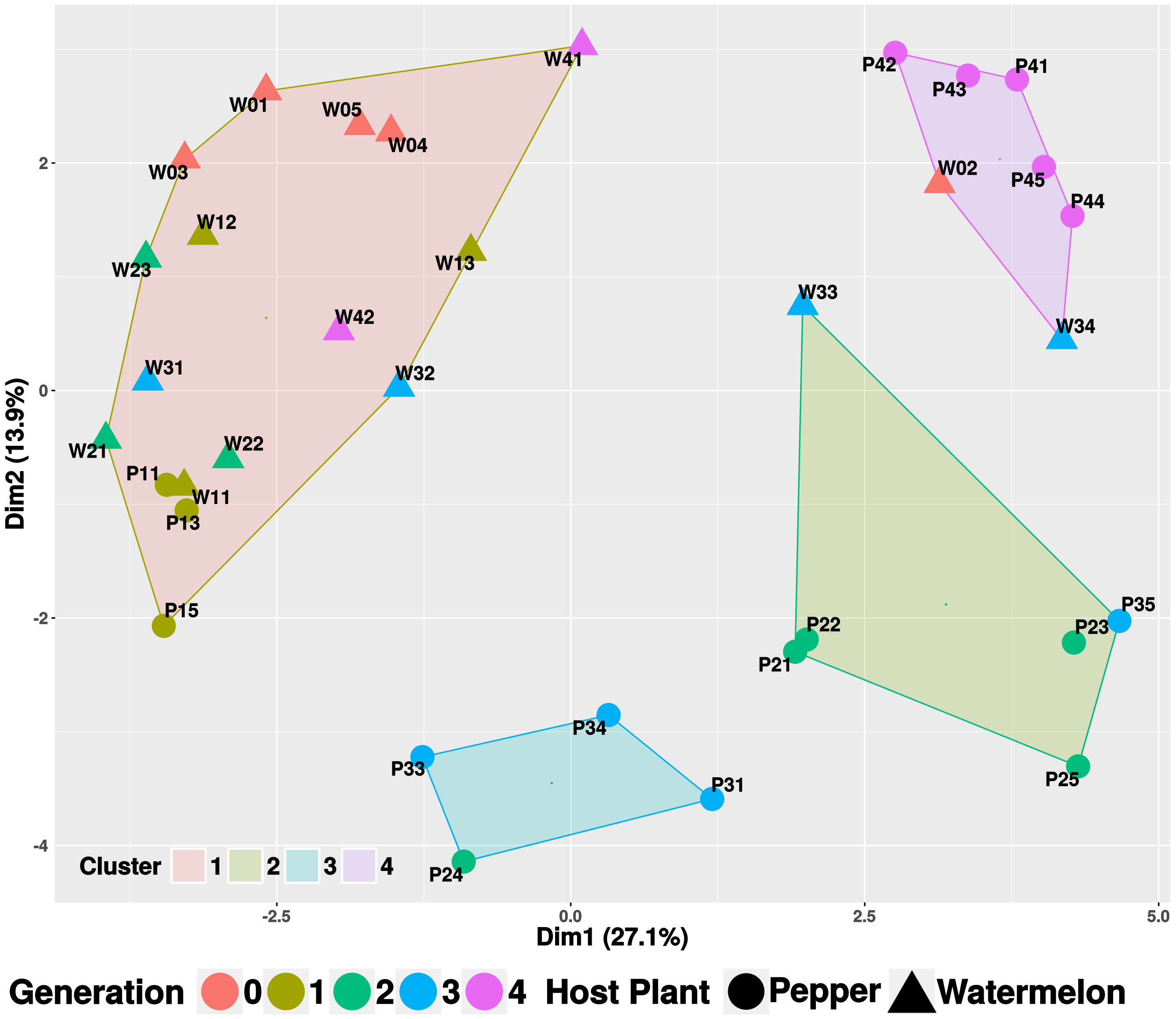

Figure S4: Partitioning Around Medoids (PAM) fine-level clustering of *B. tabaci* gut-enriched samples. Most of the watermelon samples form a single cluster together with samples of the first generation on pepper. In contrast, samples from the advanced generations on pepper form three clusters, mainly corresponding to their generational association.

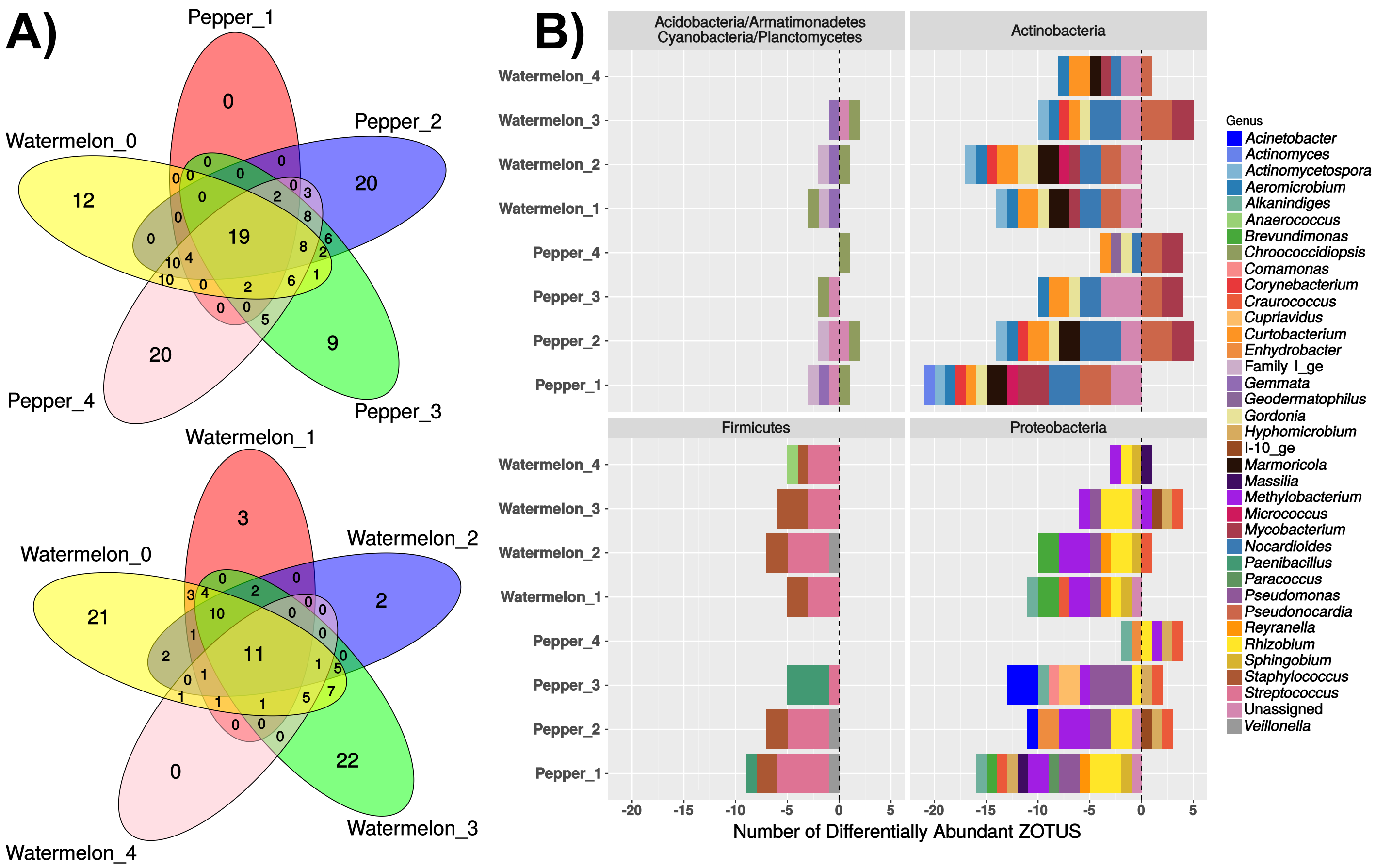

Figure S5: Averaged distribution of ZOTUs, collapsed at the genus level and grouped by host-plant and generation, when compared to the starting population. **A)** Euler diagrams showing the different averaged bacterial sets of pepper (top) and watermelon (bottom) field samples in relation to the starting population (Watermelon_0): core, shared and unique genera. **B)** ZOTUs, categorized by their genus, that show differential abundance (by DESeq2) in the starting and field populations. Negative values indicate ZOTUs that were more abundant in the starting population, positive values indicate greater abundance in the field populations.

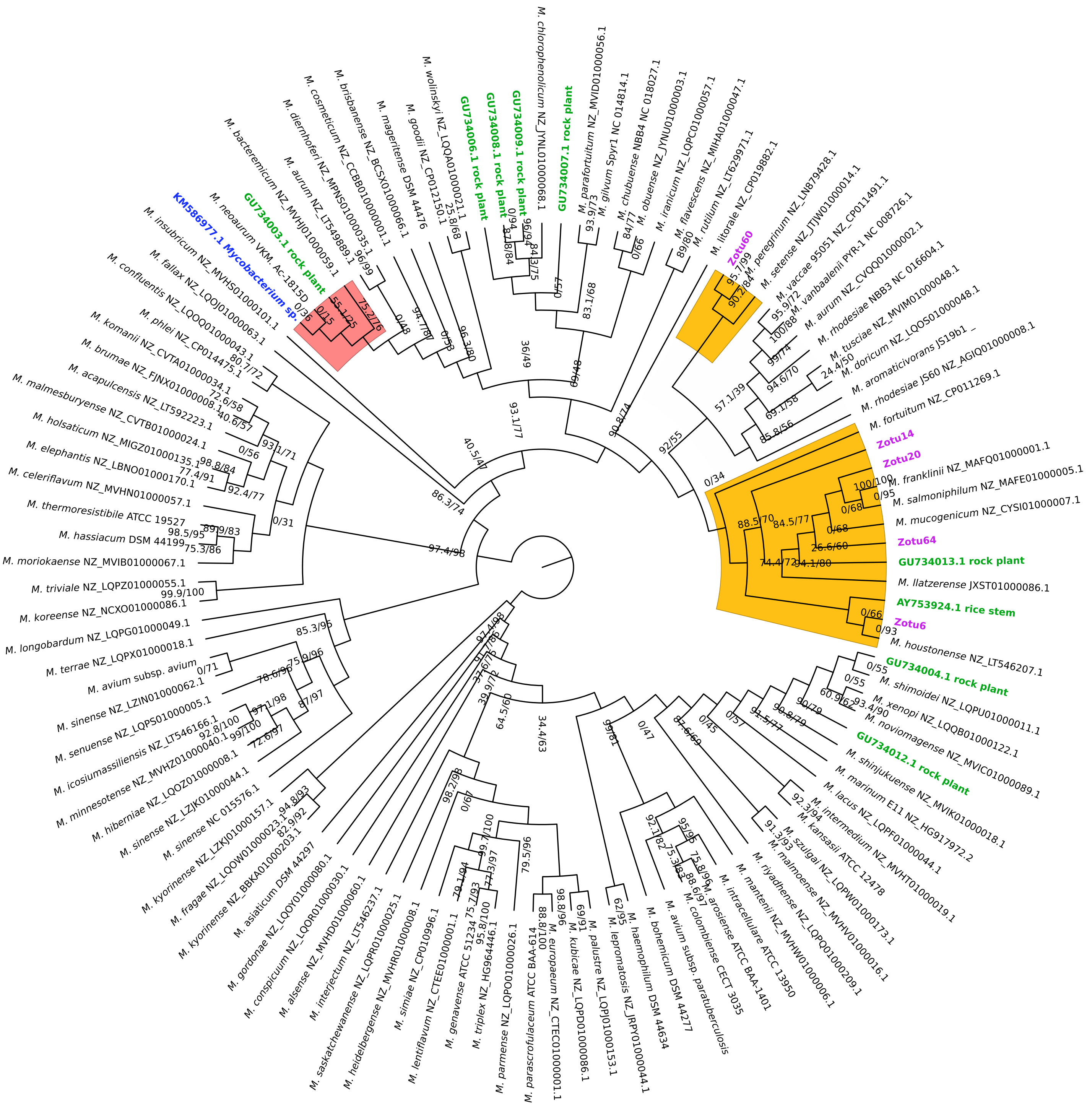

Figure S6: Phylogenetic relationship, based on the 16S rRNA gene, of the obtained *Mycobacterium* ZOTUS (purple). 16S rRNA genes from sequenced *Mycobacterium* genomes (black), partial 16S rRNA gene sequences from plant *Mycobacterium* endophytes (green) [31, 32], and one aphid-associated *Mycobacterium* (blue) [33] are presented. The maximum likelihood tree was midpoint rooted, and is presented as a cladogram for plotting reasons. Node labels show bootstrap/SH-aLRT support values. *B. tabaci*-associated *Mycobacterium* ZOTUs lineages are highlighted in orange. The aphid-associated *Mycobacterium* lineage is highlighted in pink.

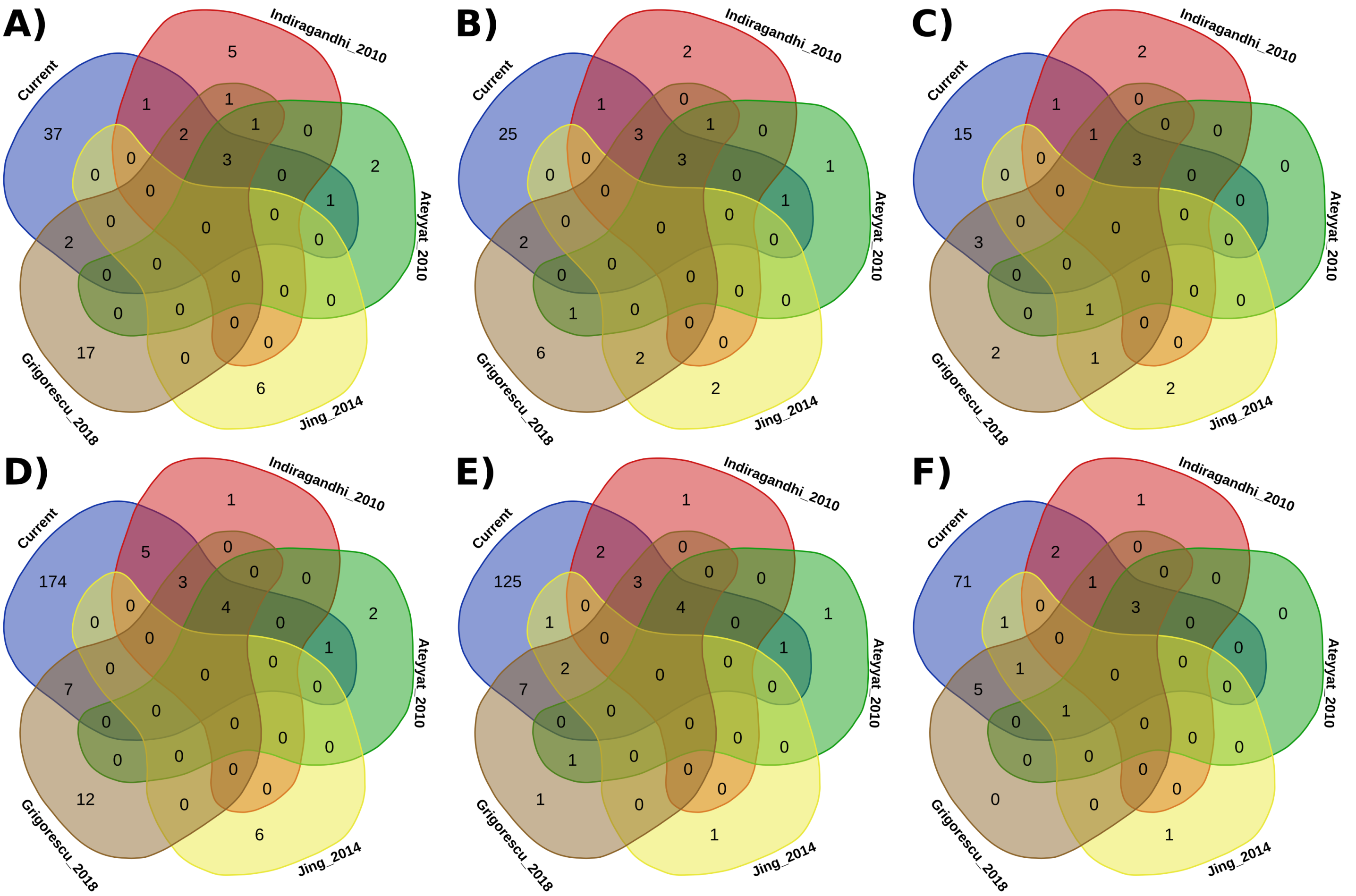

Figure S7: ZOTUs core set and full diversity (pan-microbiome) detected in the current study compared to all bacteria previously detected in *B. tabaci* MEAM1 by culture-dependent (Ateyyat_2010 [34] and Indiragandhi_2010 [35]) and culture-independent (Jing_2014 [36]) microbiomes. Euler diagrams showing the different numbers of genera (**A**, **D**), families (**B**, **E**), and orders (**C**, **F**) shared between the core set (**A-C**) or the pan-microbiome (**D-F**) and the rest of microbiomes. For comparative purposes, one aphids' microbiome study (Grigorescu_2018 [33]) is included as a non-*Bemisia* phloem-feeding insects.

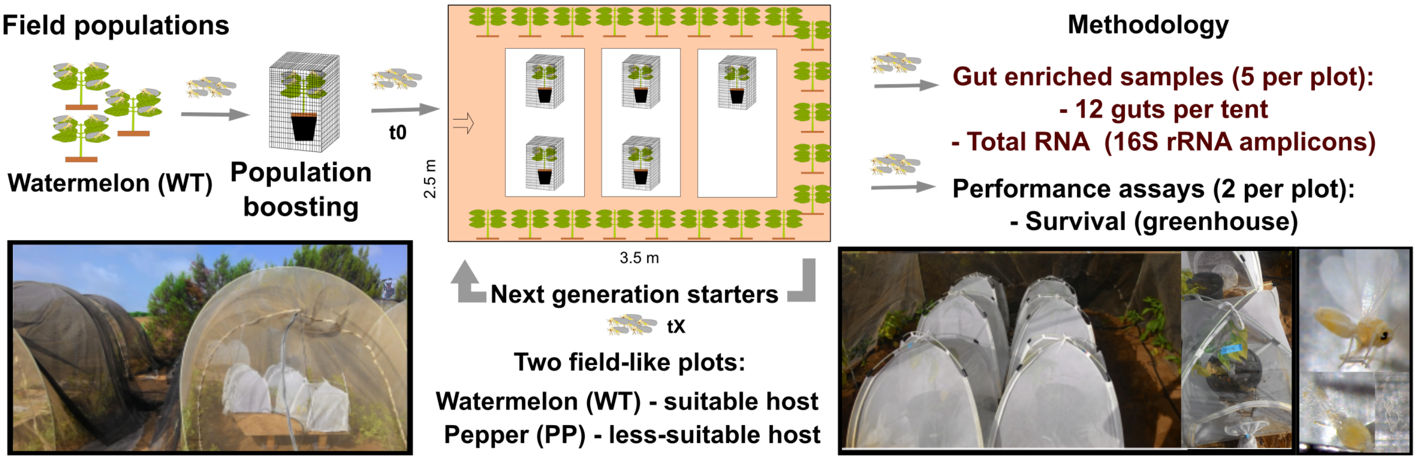

Figure S8: Schema of the field-like experiment conducted at the Hebrew University Experimental Farm in Rehovot.

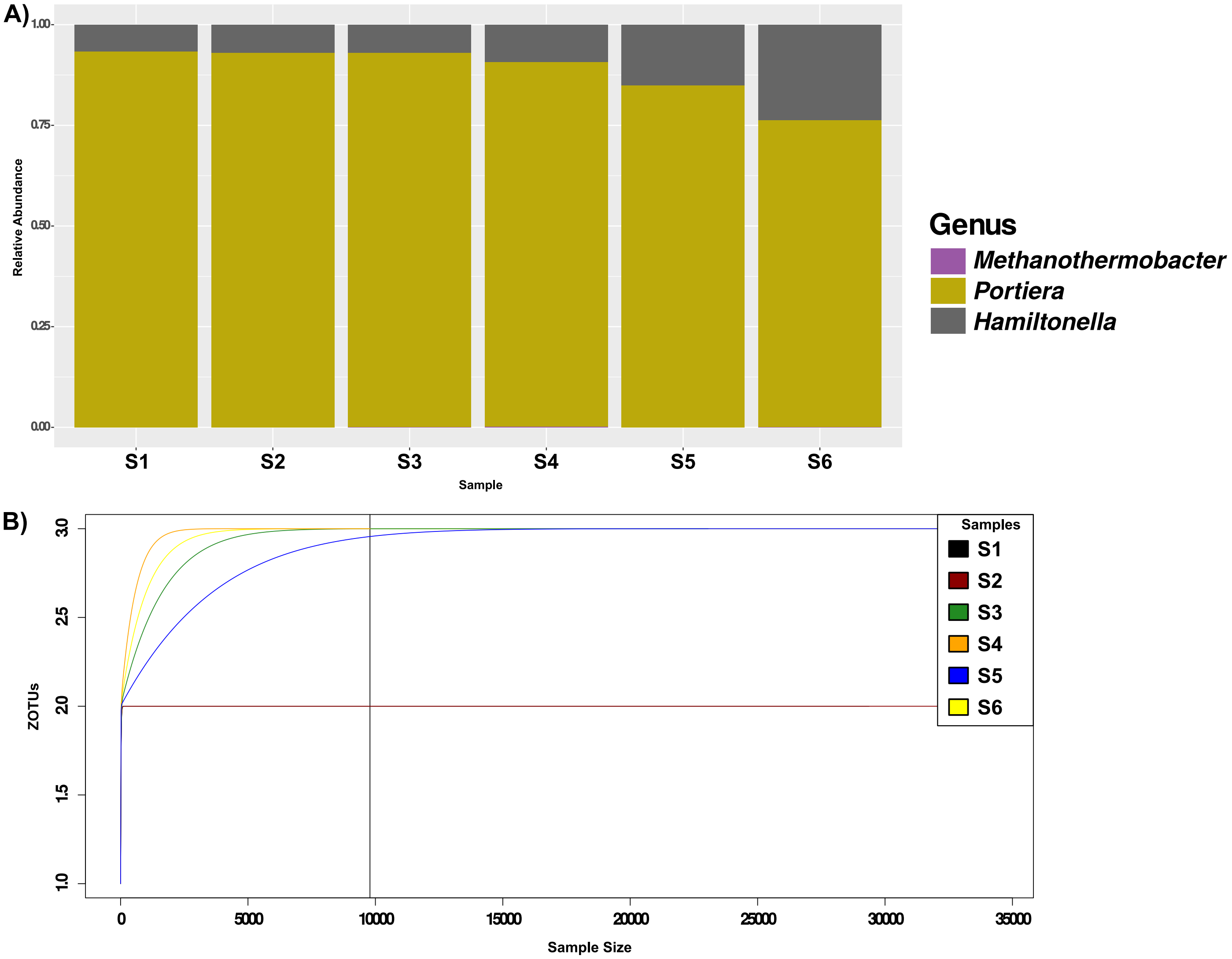

Figure S9: Miseq sequencing trial of six gut-enriched samples. **A)** Barplot and **B)** sampling effort curves. Only one ZOTU was not classified as a *B. tabaci*'s endosymbiont.

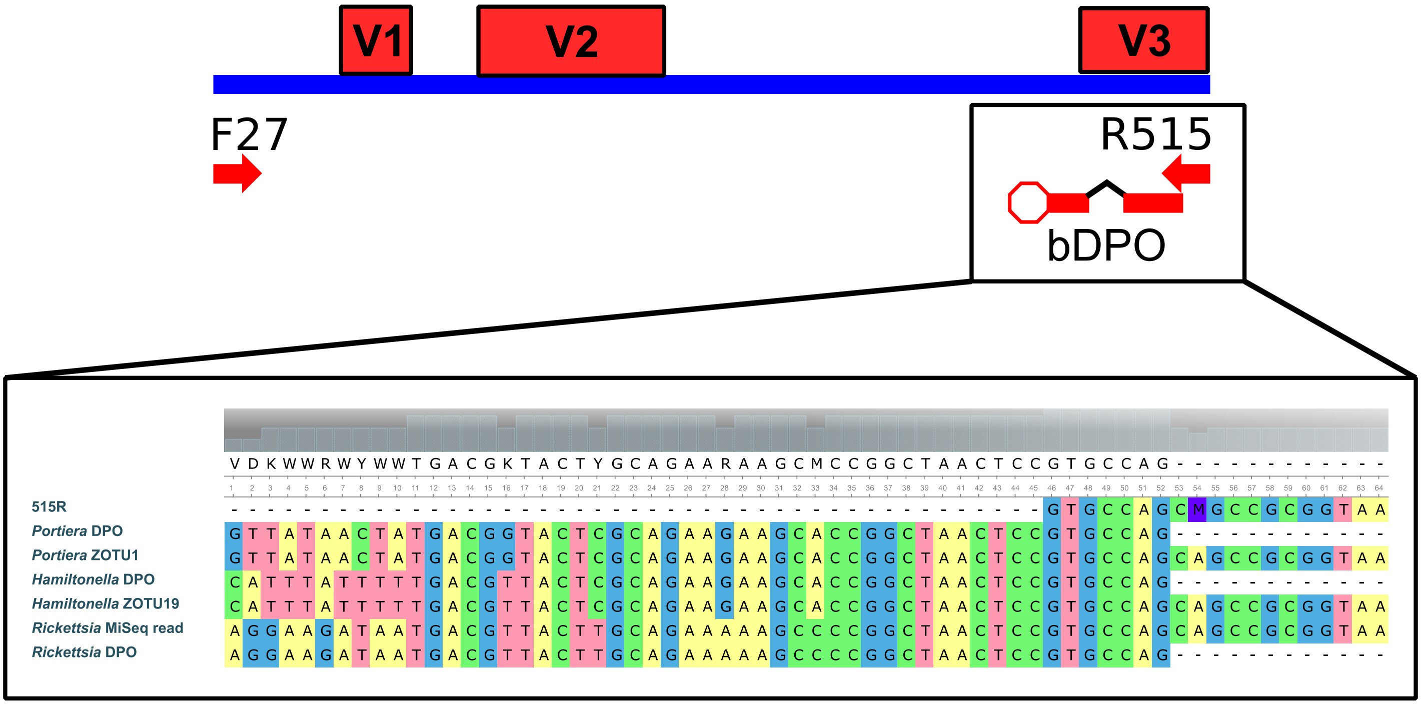

Figure S10: Schematic representation of a blocking Dual Priming Oligonucleotide (bDPO). The zoomed region shows the bDPOs alignment against the 515R universal primer and ZOTUs/sequences recovered for each endosymbiont in the trial experiment.

Supplementary Tables

Table S1: The sequenced samples, their associated metadata, and richness results with and without reads belonging to *B. tabaci* endosymbionts.

					RAW Reads			Endosymbionts			Other Bacteria					
**Sample**	**Host Plant**	**Gen.**	**Collection**	**cDNA**	**Assembled**	**Mapped**	**Mapped %**	**Reads**	**Shannon**	**Simpson**	**Reads**	**Blocked %**	**Final Reads***	**ZOTUs***	**Shannon***	**Simpson***
**W01**	Watermelon	0	26/07/15	44.96	82334	77906	94.62	72841	0.71	0.21	5065	6.5	3321	90	3.27	0.93
**W02**	Watermelon	0	26/07/15	28.64	138935	109474	78.80	31565	4.01	0.93	77909	71.17	53125	284	4.17	0.97
**W03**	Watermelon	0	26/07/15	27.2	159924	153700	96.11	146254	0.64	0.23	7446	4.84	4923	106	3	0.89
**W04**	Watermelon	0	26/07/15	62.24	205490	197576	96.15	191778	0.55	0.18	5798	2.93	4546	98	3.27	0.93
**W05**	Watermelon	0	26/07/15	32.32	185745	176648	95.10	163236	0.99	0.35	13412	7.59	12072	108	2.76	0.88
**P11**	Pepper	1	20/08/15	18.24	118577	114909	96.91	110962	1	0.52	3947	3.43	2695	68	2.96	0.9
**P13**	Pepper	1	20/08/15	13.2	160924	156220	97.08	145720	0.71	0.24	10500	6.72	7151	81	2.76	0.87
**P15**	Pepper	1	20/08/15	16.8	198637	196588	98.97	192502	0.33	0.11	4086	2.08	2413	48	2.69	0.86
**P21**	Pepper	2	14/09/15	20.96	129259	125398	97.01	118017	1.06	0.47	7381	5.89	5849	135	3.56	0.94
**P22**	Pepper	2	14/09/15	40.16	114786	111289	96.95	106773	0.64	0.21	4516	4.06	3636	125	3.79	0.96
**P23**	Pepper	2	14/09/15	50.56	288967	276328	95.63	248118	0.97	0.28	28210	10.21	23104	196	3.81	0.95
**P24**	Pepper	2	14/09/15	55.36	144237	141042	97.78	136300	0.43	0.12	4742	3.36	3061	93	3.57	0.95
**P25**	Pepper	2	14/09/15	32.48	18455	11825	64.07	0	3.43	0.9	11825	100	10760	137	3.18	0.88
**P31**	Pepper	3	12/10/15	10.8	116424	113836	97.78	102670	0.87	0.27	11166	9.81	7566	96	2.91	0.9
**P33**	Pepper	3	12/10/15	43.04	144638	141916	98.12	129822	0.68	0.19	12094	8.52	4810	100	3.23	0.91
**P34**	Pepper	3	12/10/15	10.88	346968	339191	97.76	331638	0.48	0.16	7553	2.23	5348	119	3.65	0.95
**P35**	Pepper	3	12/10/15	13.84	203025	194398	95.75	83332	3.21	0.81	111066	57.13	91377	267	3.96	0.94
**P41**	Pepper	4	15/11/15	22.4	228052	208780	91.55	125908	2.52	0.66	82872	39.69	60663	320	4	0.95
**P42**	Pepper	4	15/11/15	63.04	126186	121395	96.20	112832	0.78	0.24	8563	7.05	6492	175	3.69	0.94
**P43**	Pepper	4	15/11/15	54.08	208990	198496	94.98	185027	0.87	0.3	13469	6.79	10771	174	3.45	0.93
**P44**	Pepper	4	15/11/15	31.36	208639	194176	93.07	90044	3.01	0.8	104132	53.63	65406	231	3.74	0.95
**P45**	Pepper	4	15/11/15	110.4	185911	177401	95.42	170330	0.67	0.24	7071	3.99	5763	166	3.76	0.96
**W11**	Watermelon	1	06/09/15	33.12	37724	35855	95.05	31706	0.77	0.22	4149	11.57	3126	68	3.01	0.92
**W12**	Watermelon	1	06/09/15	49.6	77826	75685	97.25	66175	0.92	0.28	9510	12.57	5090	95	3.01	0.91
**W13**	Watermelon	1	06/09/15	135.2	94500	90005	95.24	73307	1.2	0.35	16698	18.55	13659	133	3.11	0.9
**W15****	Watermelon	1	06/09/15	29.44	205566	194006	94.38	147259	1.22	0.47	46747	24.1	40863	93	1.24	0.4
**W21**	Watermelon	2	27/09/15	90.4	19392	7651	39.45	3664	2.42	0.75	3987	52.11	2597	66	2.68	0.85
**W22**	Watermelon	2	27/09/15	26.72	152347	149441	98.09	144795	0.38	0.11	4646	3.11	3262	95	3.29	0.93
**W23**	Watermelon	2	27/09/15	30.08	86270	83956	97.32	76539	0.74	0.22	7417	8.83	4969	103	3.07	0.89
**W31**	Watermelon	3	08/11/15	15.36	91947	89428	97.26	83363	0.63	0.18	6065	6.78	3780	72	2.86	0.88
**W32**	Watermelon	3	08/11/15	26.24	173430	168021	96.88	163622	0.62	0.23	4399	2.62	3272	82	3.18	0.92
**W33**	Watermelon	3	08/11/15	14.96	181063	175508	96.93	159507	0.88	0.25	16001	9.12	12347	146	3.45	0.93
**W34**	Watermelon	3	08/11/15	15.84	62588	58836	94.01	29132	2.96	0.79	29704	50.49	19468	200	3.89	0.95
**W41**	Watermelon	4	03/12/15	37.12	225185	212295	94.28	184551	1.22	0.38	27744	13.07	18319	182	3.36	0.92
**W42**	Watermelon	4	03/12/15	27.52	82772	78390	94.71	75189	0.66	0.24	3201	4.08	1863	62	2.86	0.91
**PCR**	Blank	NA	06/12/15	2.88	14613	10491	71.79	0	3.98	0.97	10459	NA	NA	107	NA	NA
**Mock**	Mock	NA	06/12/15	50	229694	202199	88.03	6	2.6	0.91	202183	NA	NA	31	NA	NA

*After discarding low prevalence and PCR ZOTUs. **W15 passed the assembly/ZOTUs checks but was considered an outlier by its low diversity.

Table S2: Primers used for *B. tabaci* species determination and endosymbionts characterization.

**Organism**	**Gene**	**Primer name**	**Sequence (5'-3')**	**Tm (°C)**	**Product size (bp)**	**Reference**
B. tabaci	*mtCOI*	C1-J-2195<br>L2-N-3014	TTGATTTTTTGGTCATCCAGAAGT<br>TCCAATGCACT AATCTGCCATATTA	52	816	[37]
Hamiltonella	*16S*	Hb-F<br>Hb-R	TGAGTAAAGTCTGGAATCTGG<br>AGTTCAAGACCGCAACCTC	58	700	[38]
Arsenophonus	*16S*	Ars23S-1<br>Ars23S-2	CGTTTGATGAATTCATAGTCAAA<br>GGTCCTCCAGTTAGTGTTACCCAAC	53	581-803	[39]
Wolbachia	*16S*	Wol16S-F<br>Wol16S-R	CGGGGGAAAAATTTATTGCT<br>AGCTGTAATACAGAAAGTAAA	53	700	[40]
Rickettsia	*16S*	Rb-F<br>Rb-R	GCTCAGAACGAACGCTATC<br>GAAGGAAAGCATCTCTGC	58	900	[3]
Cardinium	*16S*	CFB-F<br>CFB-R	GCGGTGTAAAATGAGCGTG<br>ACCTMTTCTTAACTCAAGCCT	58	395	[41]

Table S3: 16S rRNA universal primers and used linkers.

Name	Linker	Sequence
27F (V1)	ACACTGACGACATGGTTCTACA	AGAGTTTGATCMTGGCTCAG
515R (V3)	TACGGTAGCAGAGACTTGGTCT	TTACCGCGGCKGCTGGCAC
319F (V3)*	ACACTGACGACATGGTTCTACA	ACTCCTACGGGAGGCAGCAG
806R (V4)*	TACGGTAGCAGAGACTTGGTCT	GGACTACHVGGGTWTCTAAT

*Sequencing trial.

Table S4: Blocking Dual Priming Oligonucleotides designed for this study.

Name	Sequence
*Portiera*_R	CTGGCACGGAGTTAGCCGGTGCTTCTTCTGCGAGTACCGTCATAGTTATAAC
*Portiera*_DPO_R	CTGGCACGGAGTTAGCCGGTGCTTCTTCTG**IIIII**ACCGTCATAGTTATAAC**C3**
*Hamiltonella*_R	CTGGCACGGAGTTAGCCGGTGCTTCTTCTGCGAGTAACGTCAAAAATAAATGG
*Hamiltonella*_DPO_R	CTGGCACGGAGTTAGCCGGTGCTTCTTCTG**IIIII**AACGTCAAAAATAAATGG**C3**
*Rickettsia*_R	CTGGCACGGAGTTAGCCGGGGCTTTTTCTGCAAGTAACGTCATTATCTTCCT
*Rickettsia*_DPO_R	CTGGCACGGAGTTAGCCGGGGCTTTTTCT**IIIII**TAACGTCATTATCTTCCT**C3**

I: deoxyinosine. C3: 3 carbon chain attached to the terminal 3' hydroxyl group of the last nucleotide.

References

1. Liu SS, Colvin J, De Barro PJ. Species concepts as applied to the whitefly *Bemisia tabaci* systematics: How many species are there? *J Integr Agric*. 2012 feb;11:176–186.

2. Malka O, Santos-Garcia D, Feldmesser E, Sharon E, Krause-Sakate R, Delatte H, *et al.* Species-complex diversification and host-plant associations in *Bemisia tabaci*: A plant-defence, detoxification perspective revealed by RNA-Seq analyses. *Mol Ecol*. 2018 nov;27:4241–4256.

3. Gottlieb Y, Ghanim M, Chiel E, Gerling D, Portnoy V, Steinberg S, *et al.* Identification and localization of a Rickettsia sp. in Bemisia tabaci (Homoptera: Aleyrodidae). *Appl Environ Microbiol*. 2006 may;72:3646–3652.

4. Gottlieb Y, Ghanim M, Gueguen G, Kontsedalov S, Vavre F, Fleury F, Zchori-Fein E. Inherited intracellular ecosystem: symbiotic bacteria share bacteriocytes in whiteflies. *FASEB J*. 2008 jul;22:2591–9259.

5. Brumin M, Levy M, Ghanim M. Transovarial transmission of *Rickettsia* spp. and organ-specific infection of the whitefly *Bemisia tabaci*. *Appl Environ Microbiol*. 2012 aug;78:5565–5574.

6. Zidon R, Tsueda H, Morin E, Morin S. Projecting pest population dynamics under global warming: the combined effect of inter- and intra-annual variations. *Ecol Appl*. 2016 jun;26:1198–11210.

7. Simmons AM, Levi A. Sources of whitefly (Homoptera: Aleyrodidae) resistance in *Citrullus* for the improvement of cultivated watermelon. *HortScience*. 2002;37:581–584.

8. Ogbuji K, McCutcheon1 GS, Simmons AM, Snook ME, Harrison HF, Levi A. Partial leaf chemical profiles of a desert watermelon species. *HortScience*. 2012;47:580–584.

9. Coffey JL, Simmons AM, Merle Shepard B, Tadmor Y, Levi A. Potential sources of whitefly (Hemiptera: Aleyrodidae) resistance in desert watermelon (*Citrullus colocynthis*) germplasm. *HortScience*. 2015;50:13–17.

10. Frantz JD, Gardner J, Hoffmann MP, Jahn MM. Greenhouse screening of *Capsicum* accessions for resistance to green peach aphid (*Myzus persicae*). *HortScience*. 2004;39:1332–1335.

11. Iida H, Kitamura T, Honda Ki. Comparison of egg-hatching rate, survival rate and development time of the immature stage between B- and Q-biotypes of *Bemisia tabaci* (Gennadius) (Homoptera: Aleyrodidae) on various agricultural crops. *Appl Entomol Zool*. 2009;44:267–273.

12. Tsueda H, Tsuchida K. Reproductive differences between Q and B whiteflies, Bemisia tabaci, on three host plants and negative interactions in mixed cohorts. *Entomol Exp Appl*. 2011;141(3):197–207.

13. Ballina-Gomez H, Ruiz-Sanchez E, Chan-Cupul W, Latournerie-Moreno L, Hernández-Alvarado L, Islas-Flores I, Zuñiga-Aguilar JJ. Response of *Bemisia tabaci* Genn. (Hemiptera: Aleyrodidae) biotype B to genotypes of pepper *Capsicum annuum* (Solanales: Solanaceae). *Neotrop Entomol*. 2013 apr;42:205–210.

14. Jiao X, Xie W, Guo L, Liu B, Wang S, Wu Q, Zhang Y. Differing effects of cabbage and pepper on B and Q putative species of Bemisia tabaci. *J Pest Sci*. 2014;87(4):629–637.

15. Latournerie-Moreno L, Ic-Caamal A, Ruiz-Sánchez E, Ballina-Gómez H, Islas-Flores I, Chan-Cupul W, González-Mendoza D. Survival of *Bemisia tabaci* and activity of plant defense-related enzymes in genotypes of *Capsicum annuum* L. *Chil J Agric Res*. 2015;75:71–77.

16. Lei H, Tjallingii WF, Lenteren JC. Effect of tethering during EPG recorded probing by adults of the greenhouse whitefly. *J Appl Entomol*. 1997;121:211–217.

17. Chun JY, Kim KJ, Hwang IT, Kim YJ, Lee DH, Lee IK, Kim JK. Dual priming oligonucleotide system for the multiplex detection of respiratory viruses and SNP genotyping of CYP2C19 gene. *Nucleic Acids Res*. 2007;35:e40.

18. Loy A, Arnold R, Tischler P, Rattei T, Wagner M, Horn M. probeCheck – a central resource for evaluating oligonucleotide probe coverage and specificity. *Environ Microbiol*. 2008 oct;10:2894–2898.

19. Vestheim H, Jarman SN. Blocking primers to enhance PCR amplification of rare sequences in mixed samples - a case study on prey DNA in Antarctic krill stomachs. *Front Zool.* 2008 jul;5:12.

20. Martin M. Cutadapt removes adapter sequences from high-throughput sequencing reads. *EMBnetjournal.* 2011 may;17:10.

21. Edgar RC. Search and clustering orders of magnitude faster than BLAST. *Bioinformatics.* 2010 oct;26:2460–2461.

22. Schloss PD, Westcott SL, Ryabin T, Hall JR, Hartmann M, Hollister EB, *et al.* Introducing mothur: open-source, platform-independent, community-supported software for describing and comparing microbial communities. *Appl Environ Microbiol.* 2009 dec;75:7537–7541.

23. Quast C, Pruesse E, Yilmaz P, Gerken J, Schweer T, Yarza P, *et al.* The SILVA ribosomal RNA gene database project: improved data processing and web-based tools. *Nucleic Acids Res.* 2013 jan;41:D590–D596.

24. McMurdie PJ, Holmes S. Waste not, want not: why rarefying microbiome data is inadmissible. *PLoS Comput Biol.* 2014 apr;10:e1003531.

25. Langille MGI, Zaneveld J, Caporaso JG, McDonald D, Knights D, Reyes Ja, *et al.* Predictive functional profiling of microbial communities using 16S rRNA marker gene sequences. *Nat Biotechnol.* 2013 sep;31:814–821.

26. Abubucker S, Segata N, Goll J, Schubert AM, Izard J, Cantarel BL, *et al.* Metabolic reconstruction for metagenomic data and its application to the human microbiome. *PLoS Comput Biol.* 2012;8:e1002358.

27. Segata N, Izard J, Waldron L, Gevers D, Miropolsky L, Garrett WS, Huttenhower C. Metagenomic biomarker discovery and explanation. *Genome Biol.* 2011 jun;12:R60.

28. Katoh K, Misawa K, Kuma Ki, Miyata T. MAFFT: a novel method for rapid multiple sequence alignment based on fast Fourier transform. *Nucleic Acids Res.* 2002 jul;30:3059–3066.

29. Castresana J. Selection of conserved blocks from multiple alignments for their use in phylogenetic analysis. *Mol Biol Evol.* 2000 apr;17:540–552.

30. Nguyen LT, Schmidt HA, von Haeseler A, Minh BQ. IQ-TREE: a fast and effective stochastic algorithm for estimating maximum-likelihood phylogenies. *Mol Biol Evol.* 2015 jan;32:268–274.

31. Tian X, Cao L, Tan H, Han W, Chen M, Liu Y, Zhou S. Diversity of cultivated and uncultivated actinobacterial endophytes in the stems and roots of rice. *Microb Ecol.* 2007 may;53:700–707.

32. Koskimäki JJ, Hankala E, Suorsa M, Nylund S, Pirttilä AM. Mycobacteria are hidden endophytes in the shoots of rock plant [*Pogonatherum paniceum* (Lam.) Hack.] (Poaceae). *Environ Microbiol Rep.* 2010 aug;2:619–624.

33. Grigorescu AS, Renoz F, Sabri A, Foray V, Hance T, Thonart P. Accessing the hidden microbial diversity of aphids: an illustration of how culture-dependent methods can be used to decipher the insect microbiota. *Microb Ecol.* 2018 may;75:1035–1048.

34. Ateyyat M, Shatnawi M, Al-Mazra'awi M. Isolation and identification of culturable forms of bacteria from the sweet potato whitefly Bemesia tabaci Genn.(Homoptera: Aleyrodidae) in Jordan. *Turk J Agric For.* 2010;34(3):225–234.

35. Indiragandhi P, Yoon C, Oh Yang J, Cho S, Min Sa T, Kim GH. Microbial Communities in the Developmental Stages of B and Q Biotypes of Sweetpotato Whitefly, Bemisia tabaci (Hemiptera: Aleyrodidae). *J Korean Soc Appl Biol Chem.* 2010;53(5):605–617. Available from: http://link.springer.com/10.3839/jksabc.2010.093.

36. Jing X, Wong ACN, Chaston JM, Colvin J, McKenzie CL, Douglas AE. The bacterial communities in plant phloem-sap-feeding insects. *Mol Ecol.* 2014;23:1433–1444.

37. Frohlich DR, Torres-Jerez I, Bedford ID, Markham PG, Brown JK. A phylogeographical analysis of the *Bemisia tabaci* species complex based on mitochondrial DNA markers. *Mol Ecol.* 1999 oct;8:1683–1691.

38. Zchori-Fein E, Brown JK. Diversity of prokaryotes associated with *Bemisia tabaci* (Gennadius) (Hemiptera: Aleyrodidae). *Ann Entomol Soc Am.* 2002 nov;95:711–718.

39. Thao ML, Baumann P. Evidence for multiple acquisition of *Arsenophonus* by whitefly species (Sternorrhyncha: Aleyrodidae). *Curr Microbiol.* 2004 feb;48:140–144.

40. Heddi A, Grenier AM, Khatchadourian C, Charles H, Nardon P. Four intracellular genomes direct weevil biology: nuclear, mitochondrial, principal endosymbiont, and *Wolbachia*. *Proc Natl Acad Sci U S A.* 1999 jun;96:6814–6819.

41. Weeks AR, Velten R, Stouthamer R. Incidence of a new sex-ratio-distorting endosymbiotic bacterium among arthropods. *Proc R Soc B Biol Sci.* 2003 sep;270:1857–1865.
